# Validation and characterization of a novel blood–brain barrier platform for investigating traumatic brain injury

**DOI:** 10.1038/s41598-023-43214-7

**Published:** 2023-09-26

**Authors:** Christopher T. Bolden, Max A. Skibber, Scott D. Olson, Miriam Zamorano Rojas, Samantha Milewicz, Brijesh S. Gill, Charles S. Cox

**Affiliations:** 1 Department of Pediatric Surgery, McGovern Medical School, The University of Texas Health Science Center at Houston (UTHealth), Houston, TX USA; 2 Center for Translational Injury Research, The University of Texas Health Science Center at Houston (UTHealth), Houston, TX USA; 3 Department of Surgery, McGovern Medical School, The University of Texas Health Science Center at Houston (UTHealth), Houston, TX USA

**Keywords:** Blood-brain barrier, Neurological disorders, Preclinical research

## Abstract

The Blood–Brain Barrier (BBB) is a highly-selective physiologic barrier responsible for maintaining cerebral homeostasis. Innovative in vitro models of the BBB are needed to provide useful insights into BBB function with CNS disorders like traumatic brain injury (TBI). TBI is a multidimensional and highly complex pathophysiological condition that requires intrinsic models to elucidate its mechanisms. Current models either lack fluidic shear stress, or neglect hemodynamic parameters important in recapitulating the human in vivo BBB phenotype. To address these limitations in the field, we developed a fluid dynamic novel platform which closely mimics these parameters. To validate our platform, Matrigel-coated Transwells were seeded with brain microvascular endothelial cells, both with and without co-cultured primary human astrocytes and bone-marrow mesenchymal stem cells. In this article we characterized BBB functional properties such as TEER and paracellular permeability. Our platform demonstrated physiologic relevant decreases in TEER in response to an ischemic environment, while directly measuring barrier fluid fluctuation. These recordings were followed with recovery, implying stability of the model. We also demonstrate that our dynamic platform is responsive to inflammatory and metabolic cues with resultant permeability coefficients. These results indicate that this novel dynamic platform will be a valuable tool for evaluating the recapitulating BBB function i*n vitro*, screening potential novel therapeutics, and establishing a relevant paradigm to evaluate the pathophysiology of TBI.

## Introduction

The Blood–Brain Barrier (BBB) is a highly dynamic interface that separates the brain microenvironment from the peripheral blood flow^1^. This specialized vasculature maintains brain homeostasis by selectively transporting essential molecules^2^. An uninterrupted layer of brain microvascular endothelial cells (BMECs), sealed together by tight junction proteins (TJs), form the BBB’s luminal boundary and control molecule transport by ATP binding cassette (ABC)-type efflux transporters and solute carriers^3^. Beyond the endothelium, BMECs are arranged with neurons, astrocytes, pericytes, and microglia to form the neurovascular unit (NVU), the brain’s basic anatomical and functional unit^4^. Together these neurovascular cells modulate brain homeostasis and cerebrovascular hemodynamics. Upon pathologic disruption, acute BBB dysfunction initiates prolonged inflammatory cascades creating chronic neurological deficits^5^.

In the context of traumatic brain injury (TBI), the delivery of mechanical force to the head causes BBB disruption, acute phase axonal damage, and glial cell activation^6^. These disturbances precede a more chronic cycle characterized by systemic dysregulation and cellular damage mechanisms^7^. A compromised BBB becomes passively permeable to an influx of electrolytes, leading to decreased trans capillary oncotic pressure, enhanced filtration, and vasogenic edema^8^. At the same time, the CNS’s diminished autoregulatory function permits elevated arterial inflow pressure and hydrostatic capillary pressure^9^. In conjunction, these mechanisms raise intracranial pressure (ICP) to dangerous levels, thereby, collapsing subdural venous vasculature, diminishing cerebral perfusion pressure (CPP), and compounding the risk of secondary ischemia^10^. The cycle of systemic complication and cellular injury is further perpetuated by intracellular cytotoxic edema, necrosis and/or apoptosis, neuroinflammation, metabolic distress, and synaptic dysfunction^11^. Tiered therapeutic strategies for TBI aim to attenuate secondary injury cascades and restore perfusion^6,12,13^.

While prevention of secondary brain injury and hemodynamic optimization are ubiquitous in TBI treatment guidelines, there exists little consensus on appropriate resuscitation benchmarks for ICP and CPP. Moreover, different pressure-targeted guidelines recommend widely diverse therapeutic strategies^9^. Given the clinical emphasis on hemodynamic stability but discordance in defining optimal treatment goals, further research is needed to comprehensively determine the individual contributions of pathological disturbances and therapeutic interventions on BBB integrity in the presence of varying hydraulic forces.

As many primary injuries leading to BBB failure remain unresolved after standard clinical interventions, high fidelity models are required to study underlying molecular mechanisms and resulting sequelae of pathological modification^14^. A number of previous models feature separate but connected compartments, multiple cell types, and integrated methods to measure permeability^15–22^. These models have significantly improved our understanding of the BBB, but do not fully replicate the complexity of physical, cellular, and chemical influences affecting BBB permeability.

An ideal in vitro TBI model would mimic the disease’s pathology by sequentially imposing the mechanical, metabolic, and inflammatory insults on a functional NVU in a high-throughput manner. The model should allow the brain and blood compartments’ hydraulic forces, perfusion rates, and biochemical environments to be independently modulated, permitting interrogation of each variable in the Starling equation^6^. The Starling equation, which can calculate the force exerted on the BBB, is an important consideration for experimentally modeling platforms TBI. Barrier function could be monitored with real-time measurements, while cells remain amenable to post-hoc analysis, such as immunohistochemistry and flow cytometry^23,24^. Finally, luminal perfusion of whole blood would enhance the model’s physiological relevance and introduce prominent intravascular interactions for study^24,25^.

**Figure 1:**
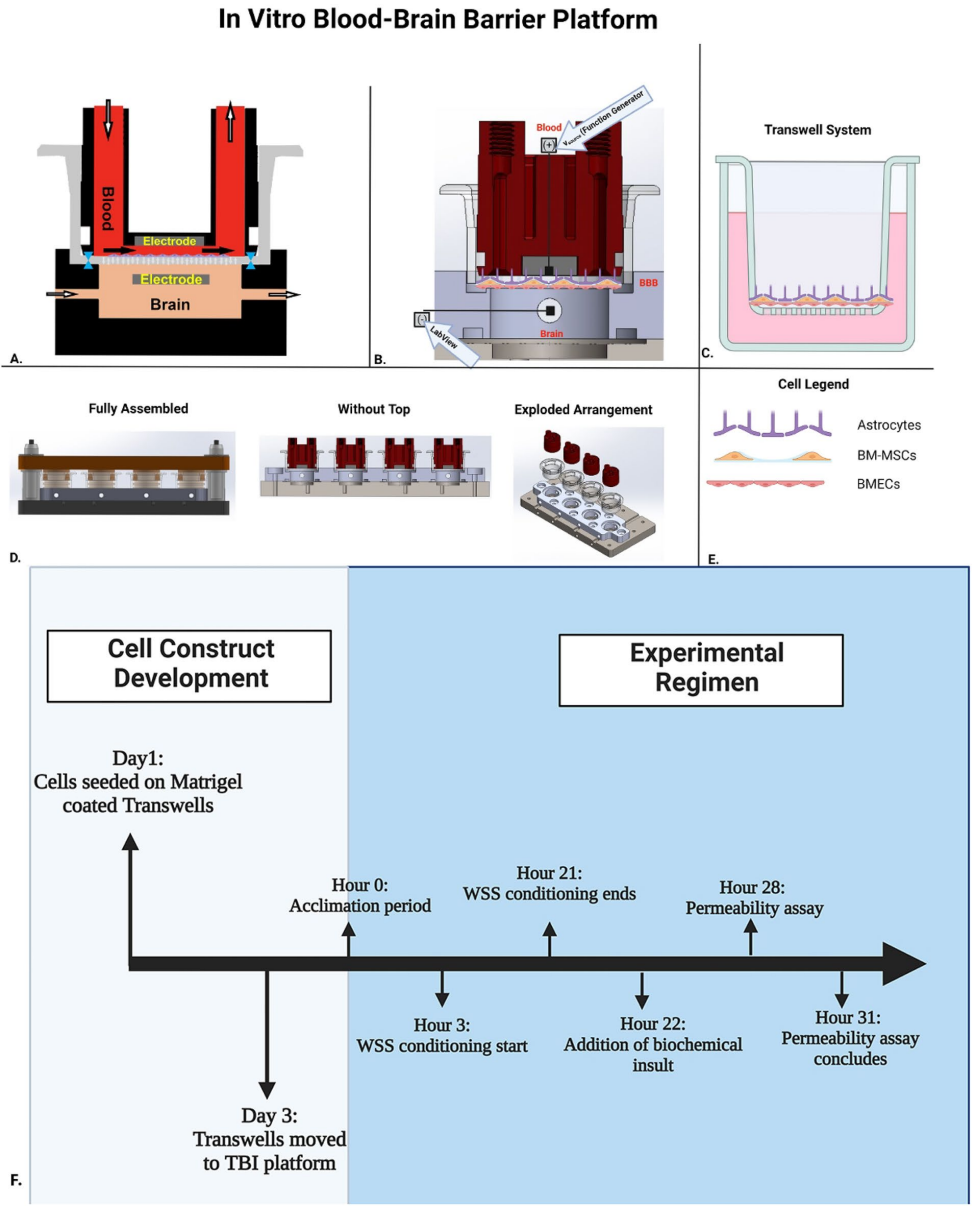
Schematic panel preparation for validation of the in vitro model platform. (**A**) Model set-up of Matrigel coated BBB Transwell in our TBI platform. Arrows represent the direction of fluid flow between or through the blood and brain compartments. Both compartments of the BBB Transwell contain an embedded aluminum electrode for measuring the electrical properties of the cultures. Pressurized seals represented in blue lock the Transwell into the platform for acclimation and measurement. (**B**) Measurements for TEER Validation of platform. A function generator produces an alternating current signal with an amplitude of 20 mV and a frequency ranging from 10 Hz to 125 kHz. The impedance spectra are recorded using an analog interface module and LabView. TEER analysis is performed in LabView where a fit of the experimental data over the frequency range 3–100 kHz is used to calculate membrane resistance (R_mem_). (**C**). Transwell system utilized for in vitro platform operations. (**D**). Dynamic platform displayed throughout three distinct assemblies: fully assembled platform, without the top, and an exploded arrangement to display each individual component. (**E**). Legend of cells that constitute tricultured BBB model in Transwell system and platform. (**F.**) Experimental timeline of validation studies. After sub culturing the cell lines for several days, HUVECs and HBEC-5is were seeded on the apical side of Matrigel coated Transwells and placed inside an incubator (37 °C and 5% CO_2_). After 3 days, Transwells were immediately transferred to our modular platform where TEER values were recorded every hour. After 3 h of flow, wall shear stress was applied for a period of 18 h. Biochemical insults are applied for an average of 6 h and permeability was assessed with different size dextrans to assess paracellular transport. Primary human astrocytes and BM-MSCs (not shown in diagram) are seeded on the apical side of Transwells on day 4 and 5, respectively and allowed to grow for 2 days. *This figure was generated from BioRender.com.*

Based on these design goals, we engineered a modular, dynamic platform around a Matrigel-coated Transwell membrane seeded with either an endothelial cell (EC) monolayer, a direct coculture with primary human astrocytes (PHA), or a triculture involving bone-marrow derived mesenchymal stem cells (BM-MSCs) (Fig. 1). To increase the physiological relevance of our triculture system, the BBB endothelium (HBEC-5i cells) were seeded in direct contact PHAs and BM-MSCs. The inductive effects of astrocytes and BM-MSCs and their roles in BBB maintenance have been well established; however, many of the models used for in vitro BBB permeability screening do not consider the direct contact the different cell types have with one another in vivo. By seeding astrocytes, BM-MSCs, and the endothelium directly atop one another this model better mimics the proximity in the NVU.

To further our physiological refinement, we developed sealed, pressurized compartments form around each face of the membrane, creating distinct blood/brain environments within a closed system. A peristaltic pump perfuses the blood luminal cavity in a pulsatile fashion to impose wall shear stress (WSS), a mechanical stimulus critical to TC expression and EC polarization^18,26–28^. Downstream variable resistive elements modulate capillary hydrostatic pressure independent of flow rate, whereas the abluminal (brain) compartment’s pressure, analogous to ICP, is altered by fluid volume. In-line pressure transducers enable real-time recording of compartmental hydraulic forces. Embedded electrodes facilitate the measurement of Transendothelial electrical resistance (TEER) via impedance spectroscopy^29^, while both compartments remain accessible for experimental assessment. Additional reservoirs and specialized tubing allows for the determination of transvascular fluid flux, an important variable of the Starling equation.

As the NVU is composed of multiple constituents, vascular and parenchymal cell populations may be cultured in their appropriate compartments. Individual BBB units may be studied as distinct elements of the cerebral microvasculature or serially linked to evolve a heterogeneous, hierarchical circuit representative of the pre-, mid-, and post-capillary zones^23,24,30^.

In this paper we describe the validation of our novel BBB platform using a combination of EC cultures. Validation objectives focused on reliably imposing the mechanical forces and biochemical insults relevant to TBI pathogenesis. Therefore, EC monolayers were exposed to WSS (average of 4 dyne/cm^2^), tumor necrosis factor alpha (TNF-α, 50 ng/ml), or an oxygen-glucose depleted environment followed by reperfusion (OGD/R). We found that our platform represents a translational relevant in vitro model to study inflammatory, metabolic, and hemodynamic changes affect the integrity of the brain microvasculature. Our platform represents a significant iteration on current in vitro models investigating TBIs as our model incorporates the application of shear stress, is designed for the addition of capillary pressure gradients to measure hydraulic conductivity, directly measures fluid flux across the BBB, and allows for post-hoc analysis of cells utilized in the platform.

## Materials and methods

### Compliance and regulations

All method were in compliance with the University of Texas Health Science Center at Houston Institutional Biosafety Committee (IBC-19-003), the institutional committee designating the use of all biological agents. All cells utilized in this study were obtained from commercially available sources.

### Reagents

24 mm Transwell^®^ with 0.4 µm Pore Polyester Membrane Inserts were obtained from Corning. HUVECs were obtained from Clonetics (San Diego, CA, USA). Immortalized HBEC-5i were obtained from American Type Culture Collection (Old Town Manassas, VA, USA). Primary human astrocytes were obtained from ScienCell Research Laboratories (Carlsbad, CA, USA). Bone marrow mesenchymal stem cells (BM-MSCs) were received as a gift from the Olson lab group^31^. Matrigel Matrix basement membrane was obtained from Corning (Bedford, MA, USA). Recombinant human TNF-α was purchased from Thermo Fisher (Minneapolis, MN, USA). Fluorescein isothiocyanate (FITC)-conjugated dextran (MW: 4,000) was purchased from Sigma Aldrich (St. Louis, MO, USA). Fluorescein isothiocyanate (FITC)-conjugated dextran (MW: 4,000) was purchased from Thermo Fisher (Minneapolis, MN, USA). Alexa Fluor 680-conjugated dextran (MW: 10,000) was purchased from Sigma Aldrich (St. Louis, MO, USA) Cytochalasin D was purchased from Sigma Aldrich (St. Louis, MO, USA). Rabbit polyclonal anti-Zonula occludens-1 (ab216880), rabbit polyclonal anti-Claudin-5 (ab15106), rabbit monoclonal (ab216327), and goat anti-rabbit (ab150077) antibodies were purchased from Abcam (Cambridge, United Kingdom).

### Cell culture

HUVECs were cultured and maintained in Endothelial Growth Medium-Plus (EGM-PLUS Medium) supplemented with endothelial growth supplement (EnGS), L-glutamine, ascorbic acid, hydrocortisone hemisuccinate, human epidermal growth factor (hEGF), heparin, fetal bovine serum (FBS), and gentamicin/amphotericin-B obtained from Lonza. HBEC-5i were initially cultured and maintained in Gibco Dulbecco’s Modified Eagle Medium: Nutrient Mixture F-12 (DMEM/F-12) with 10% FBS, 40 µg/mL endothelial growth supplement (ECGS) and 1% penicillin/streptomycin (P/S; ScienCell Research Laboratories, Inc., Carlsbad, CA, USA) in 75 cm^2^ flasks coated with 0.1% gelatin. Both cell types were incubated at 37 °C under humidified 5% CO_2_ until they respectively reached confluence. HBEC-5i and HUVECs were used between passages 4–6. PHAs were expanded and maintained in Astrocyte Medium (AM), which contains 500 mL of basal medium, 10 mL of FBS, 5 mL of astrocyte growth supplement (AGS), and 5 ml of P/S in 75 cm^2^ flasks containing a solution of poly-L-Lysine (2 μg/cm^2^). PHA were used between passages 3–6. Bone marrow derived MSCs (BM MSCs) were isolated from commercially available fresh human bone marrow aspirates from a 28 yr old male (AllCells, Alameda, CA) using density centrifugation and plastic adherence as previously described. An adherent population of MSCs was obtained 3 weeks after the initiation of culture. The cells were screened for typical spindle-like morphology and growth kinetics. The cells were further expanded by plating 10^6^ passage 2 cells at 200 cells/cm2 in 2528 cm^2^ in Nunc™ Cell Factory™ Systems with complete culture medium (CCM) that consisted of α-minimal essential medium (α-MEM; Life Technologies, Grand Island, NY), 17% fetal bovine serum (FBS; Atlanta Biologicals, Norcross, GA), 100 units/ml penicillin (Life Technologies, Carlsbad, CA), 100 μg/ml streptomycin (Life Technologies, Carlsbad, CA), and 2 mM L-glutamine (Life Technologies). At 70% cell confluency, the medium was discarded, the cultures were washed with phosphate-buffered saline (PBS) (Life Technologies, Carlsbad, CA), and the adherent cells harvested with 0.25% trypsin (Life Technologies, Carlsbad, CA) for 5 min at 37 °C and frozen at 10^6^ cells/ml for subsequent experiments as passage 3 cells. All MSC used exhibited typical plastic adherence, morphology, and phenotype consistent with the ISCT consensus definition of “MSC” (negative for CD34, CD45, CD19, and HLA-DR, positive for CD44, CD73, and CD90). BM-MSCs were used between passages 3–5.

#### HUVEC mono-culture

HUVEC were seeded at confluence onto the luminal side of Matrigel-coated Transwell inserts (0.4-µm pore size, 6-well; Corning) at a density of 2.55 × 10^5^ cells per well. 2 ml of EGM-Plus was added into the abluminal side. Monocultures were allowed to grow 48–72 h under standard conditions (maintained in a humidified incubator at 37 °C, 5% CO_2_) before acclimation to the platform. Media was exchanged every 48 h to ensure optimal cell growth.

#### HBEC-5i mono-culture

HBEC-5i cells were seeded at confluence onto the luminal side of Matrigel-coated Transwell inserts (0.4-µm pore size, 6-well; Corning) at a density of 2.55 × 10^5^ cells per well. 2 ml of EGM-Plus was added into the abluminal side. All monocultures were allowed to grow 48–72 h under standard conditions (maintained in a humidified incubator at 37 °C, 5% CO_2_) before acclimation to the platform. Media was exchanged every 48 h to ensure optimal cell growth.

#### Contact coculture

On day 1, the luminal side of the Transwell inserts were coated with Matrigel (250 μL). A 250 μL cell suspension (2.55 × 10^5^) of HBEC-5i in endothelial cell medium (ECM) (ScienCell) was added to the luminal side with 2 ml of AM (ScienCell) added to the abluminal side. On day 3, media was removed from both compartments, and 250 µL of PHA cell suspension (1.45 × 10^5^) in AM were added to the luminal compartment while, 2 mL of AM was added to the abluminal side.

#### Contact triculture

On day 1, the luminal side of the Transwell inserts were coated with Matrigel (250 μL). A 250 μL cell suspension (2.55 × 10^5^) of HBEC-5i in ECM was added to the luminal side with 2 ml of AM added to the abluminal side. On day 3, media was removed from both compartments, and 250 µL of PHA suspension (1.45 × 10^5^) in HBEC-5i medium were added to the luminal compartment while, 2 mL of ECM was added to the abluminal side. On day 5, a 100 μL (5.0 × 10^4^) cell suspension of BM-MSCs were added to the luminal side of the Transwell in MSC medium (ScienCell).

### Immunocytochemistry

Lab-tek II chamber slides (Thermo Fisher Scientific, Waltham, MA) were coated with attachment factor solution for 30 min. After 30 min of incubation and aspiration, HBEC-5i were seeded. After 48 h, PHAs and BM-MSCs were seeded. Cells were fixed with 8% paraformaldehyde (PFA) (− 20 °C) for 15 min at 37 °C in 5% CO_2_ and then 4% PFA at RT. Slides were washed with 1X PBS 2 × for 5 min and incubated with blocking solution (10% Donkey serum [Sigma, D9663] for 1 h. Cultures were incubated with primary antibodies overnight. Secondary antibodies were incubated for 2 h at RT. The following antibodies were used: anti-claudin-5 (Thermo Fisher, 35–2500), anti-occludin (Thermo Fisher, 33–1500), anti-zo1(Thermo Fisher, 61–7300), goat anti-rabbit Alexa Fluor 488 (Thermo Fisher, A11008), and goat anti-rabbit Alexa Fluor 555 (A21428, Thermo Fisher). Cells were imaged with a Leica DFC3000 G.

### Transwell immunocytochemistry

Confluent Transwells were fixed with 8% paraformaldehyde (PFA) (− 20 °C) for 15 min at 37 °C in 5% CO_2_ and then 4% PFA at RT. Transwells were washed with 1X PBS 2 × for 5 min and incubated with blocking solution (10% Donkey serum [Sigma, D9663], 10% BSA and 2% Triton-X) for 1 h. Antibodies were diluted in combinations of blocking buffer. Transwells were incubated with primary antibodies overnight. Secondary antibodies were incubated for 2 h at RT. Transwells were washed with 1X PBS 2 × for 5 min and incubated with DAPI for 5 min. Membranes were carefully removed and placed on a microscope slide. The following antibodies were used: anti-occludin (Thermo Fisher, 33–1500), anti-zo1(Thermo Fisher, 61–7300), mouse anti-GFAP (BioLegend, 644,701), mouse anti-VE Cadherin (ThermoFisher, MA5-32,940), goat anti-rabbit Alexa Fluor 488 (Thermo Fisher, A11008), and goat anti-rabbit Alexa Fluor 555 (A21428, Thermo Fisher).

### Western blot

Cell-seeded Transwells were removed from the dynamic platform and washed with ice-cold PBS to remove excess cell media. 300 uL RIPA Lysis buffer with 1 × Halt Cocktail Protease Inhibitor (ThermoFisher) was added to the Transwell on ice for 30 min. Samples were diluted with 4 × LDS sample buffer (Invitrogen, Carlsbad, CA), 1 × reducing buffer and then boiled at 85 °C for 10 min. Each sample was loaded into the wells of a NuPAGE Novex 4–12% Bis–Tris gel (Invitrogen, Carlsbad, CA) for gel electrophoresis. Proteins were transferred onto nitrocellulose membranes. Membranes were blocked for 1 h at room temperature with either 5% BSA in TBST. Membranes were then incubated with either occludin (1:50,000), claudin-5 (1:5000), or GAPDH (1:10,000) in their respective blocking buffer for approximately 2 h at 4 °C. After primary antibody incubation, membranes were washed with TBST and incubated with secondary antibody goat anti-rabbit HRP (ThermoFisher) for 1 h at room temperature.

The following antibodies were used: anti-claudin-5 (Thermo Fisher, 35–2500), anti-occludin (Thermo Fisher, 33–1500), anti-zo1(Thermo Fisher, 61–7300), goat anti-rabbit Alexa Fluor 488 (Thermo Fisher, A11008), and goat anti-rabbit Alexa Fluor HRP (A21428, ThermoFisher). Original blots are included as supplemental Figs. 8–10. Blots are included as appear on ChemiDoc for visualization. Edges of membrane blots were cut prior to hybridization with antibodies for in-laboratory referencing and record-keeping.

### Transendothelial electrical resistance measurement

Transendothelial electrical resistance (TEER) measured changes in monolayer integrity. Both compartments of the BBB well contained an embedded aluminum electrode for measuring the electrical properties of the cultures. A function generator (Agilent 33220A, Santa Clara, CA, USA) produced an alternating current (AC) signal with an amplitude of 20 mV and a frequency ranging from 10 Hz to 125 kHz. The impedance spectra were recorded using an analog interface module and LabView 2019 (National Instruments USB-6229, Austin, TX, USA). Each recording included seven reads and was sampled at 250 kS/s. TEER analysis was performed in.

LabView where a fit of the experimental data over the frequency range 3–100 kHz was used to calculate membrane resistance (R_mem_). The LabView program was independently validated by comparing its impedance spectrum against the impedance of an electrical equivalent circuit model and cellular constructs as measured by a hardware vector analyzer (Hewlett Packard 4800A, Palo Alto, CA, USA). The measured R_mem_ was normalized for the well surface area (9.5 cm^2^) to determine the TEER (Ωcm^2^).

### Wall shear stress

As described previously^32^, Ficoll (Sigma Aldrich, St. Louis, MO, USA), was added to EGM-Plus medium at a final concentration of 5% to mimic the viscosity of peripheral blood. To produce shear stress equivalent to that of physiological conditions in vivo (4–23 dyne/cm^2^), EGM-Plus media supplemented with Ficoll was perfused into the platform through a peristaltic pump. This produced a pressure waveform similar to that seen in capillary beds (2–5 mmHg). The pressure waveform is a function of the peristaltic pump’s roller configuration and rotor speed (rpm). Pump output is a high frequency, low amplitude wave that approximates the capillary bed frequency and pulse pressure. Pressure in the capillary and interstitial compartments reached equilibrium during the acclimation phase of incubation. The platform also has an O-ring to prevent leaks and completely separates the luminal and abluminal chambers. To maintain a stable microenvironment, gas-permeable reservoirs were used to exchange O_2_ and CO_2_.

### TNF-α treatment

Tumor Necrosis Factor (TNF)—α was used to study effects on permeability and TEER. TNF-α stock solution (1 mg/mL) was made in dimethyl sulfoxide (DMSO) from Sigma Aldrich (St. Louis, MO, USA) and diluted in EGM-Plus media before addition (20 ug/ml). The final concentration of DMSO was adjusted to 0.01% to avoid possible non-specific effects. Monoculture and cocultures were treated with 50 ng/mL for 6 h. Tricultures were treated with 50, 100, & 150 ng/ml for 6 h. This concentration was selected because of its relevance in inducing changes in barrier properties as demonstrated in similar studies^33,34^.

### OGD/R

After acclimation to the platform, cultures were maintained using EGM-PLUS Medium. EGM-PLUS media was replaced with glucose-free DMEM for OGD conditioning after acclimation in the platform (Max TEER). To remove oxygen from media, media was degassed inside of a tissue culture hood dedicated for platform operation. Glucose-free DMEM was placed in a cuum bell jar and suctioned until no bubbles were visibly present. Media was flushed with anoxic gas mixture for 15 min and immediately placed in an anaerobic pouch until use. Cells were allowed to grow in a hypoxic environment (5% CO_2_ and 95% N_2_) for 4 h (After Insult) with all gas exchange intervals capped. OGD conditioning was terminated by removing glucose-free media and supplementing with EGM-PLUS media. Normal culture conditions were then returned, and cells recovered for 24 h (Recovery).

### Dextran permeability assay

Permeability assays were performed using 4 kDa fluorescein-isothiocyanate dextran (ThermoFisher), 10 kDa Alexa Fluor 680, and 40 kDa Tetramethyl dextran (ThermoFisher) in triculture as described previously^35^. All models were assessed in 15 kDa fluorescein-isothiocyanate dextran. After the period of experimental observation concluded, FITC dextran was added to the luminal side to evaluate paracellular transport. The permeability coefficient (*P*_app_) in the abluminal side was measured by a microplate reader at an excitation of 490 nm and an emission of 520 nm. Calculations for permeability coefficients are as described below:

$$P_{app} = (\left(Concentration\,of\,dextran \times volume)\div period\,of\,observation\,in\,seconds\right))\div \left(Transwell\,surface\,area\times Dextran\,concentration\right)$$

### Hydraulic conductivity assay

Tricultured inserts were acclimated in the platform for a total of 22 h. before imposing target pressure and TNF-α (100 ng/ml). At 22 h, reservoirs were attached to the platform to collect excess media accumulating from the abluminal side of the platform. After 6 h of TNF-α insult and target pressure exposure, the volume of media displacement in the reservoir was measured. Calculations for fluid flux (J_v_) and hydraulic conductivity (L_p_) were performed as described below.

Fluid flux across the triculture for the 6 h period was calculated by dividing the volume displaced by unit of time in seconds of 6 h.

$${J}_{v=mL/s}$$

To determine the effect of luminal hydrostatic pressure on transendothelial water flux, the hydraulic conductivity was calculated at different pressure intervals. Fluid flux across the cellular layer was divided by 6 h interval of time and multiplied by the known surface area of the Corning tissue culture inserts (4.67 cm^2^).

$$Lp={J}_{v}/({{\rm cm}}^{2}*{\rm cm} {H}_{2}O)$$

### Data analysis

The sample size (*n*) of each experimental group is described in each corresponding figure legend. Unless otherwise noted, experiments were repeated in three independent cohorts performed on different dates with 8 biological replicates. Each run was evaluated for significant variation to evaluate the reproducibility and robustness of the model prior to combining the biological replicates. For statistical analyses, data were expressed as means ± SD and analyzed by Student's *t* test or two-way analysis of variance (ANOVA) with Tukey's or Newman–Keul post hoc tests. Data were analyzed using GraphPad Prism software (GraphPad Software Inc., La Jolla, CA, USA). Results were considered significant at *P* < 0.05. All immunostaining images were arranged using Image J (https://imagej.nih.gov/ij/).

## Results

### Cell Surface expression, tight junction protein expression, and cell interactions

**Figure 2:**
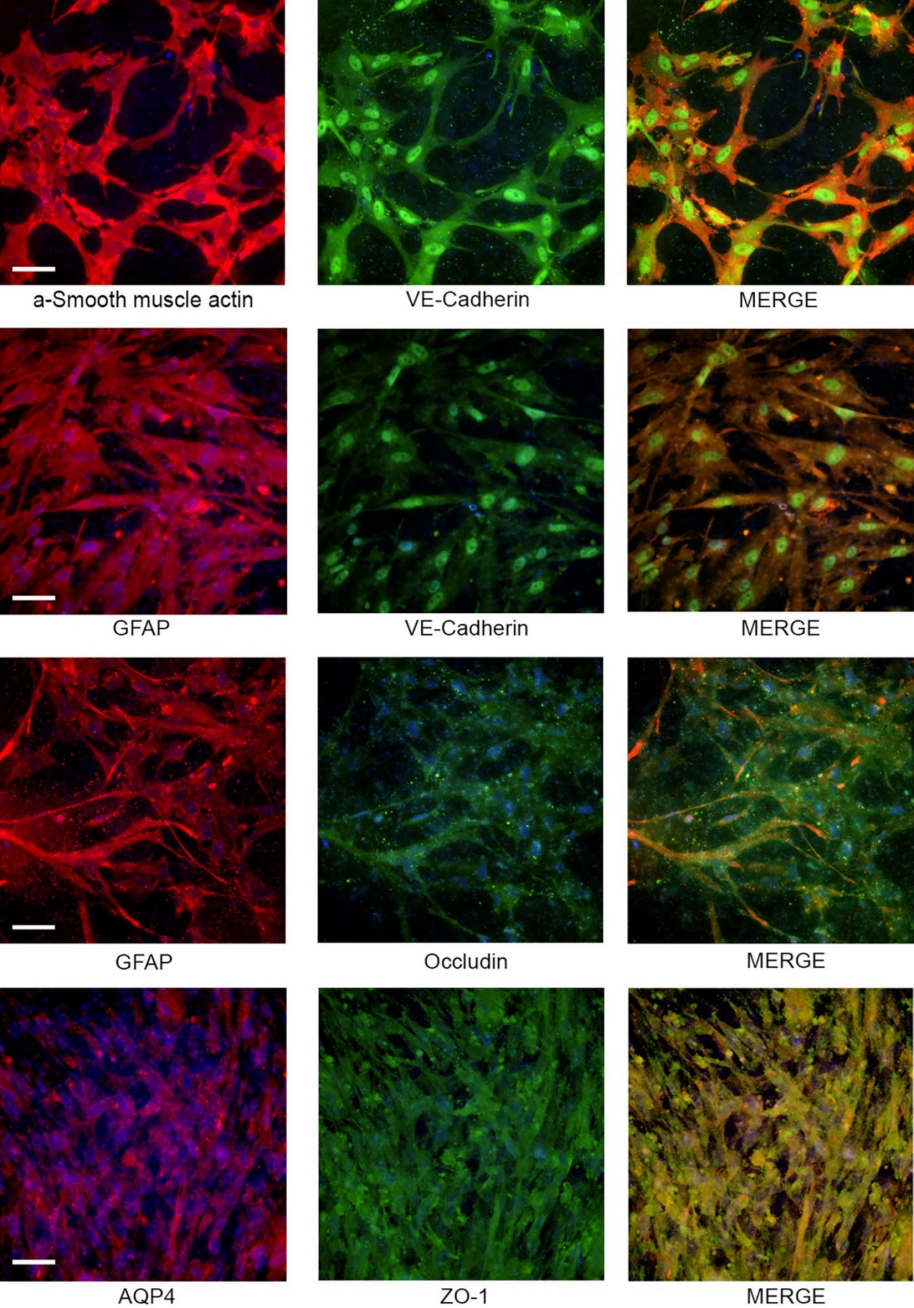
Immunostaining of contact triculture BBB model. Fluorescent cell marker expression of triculture model components including HBEC-5is (VE-cadherin, Green), primary human astrocytes (GFAP, Red), and BM-MSCs (α-smooth muscle actin, red). DAPI was used to stain nuclei of all cells in Transwell insert. Scale bar represents 100 µm.

The expression of cell surface markers was evaluated for each cell (VE-Cadherin for endothelial cells, GFAP for astrocytes, and α-SMA for pericytes/MSC) of the contact triculture (Fig. 2). Uniform GFAP expression was demonstrated across the cell layer. Immunostaining for GFAP (astrocytes) revealed interactions among the monolayers of HBEC-5i cells and BM-MSCs. Expression of α-SMA was consistent with expression of BM-MSCs. α-SMA staining of the BM-MSCs demonstrated interactions between HBEC-5i and astrocyte populations in triculture. Proteins expressed in tight junctions (occludin, claudin-5) were evaluated with Western Blot (SFig. 1A). CellTracker dye staining was performed to demonstrate cellular interactions (SFig. 1B-C) among the cell types of the triculture. CellTracker CMFDA stain astrocytes demonstrated uniform association with HBEC-5i (unlabeled) and Cell Tracker CMFPTX stained BM-MSC cells. Further staining of tight junction associate proteins was visualized in the triculture over different conditions using immunohistochemistry for ZO-1 and Claudin-5 (SFig. 2).

### Evaluation of coculture and wall shear stress on transendothelial electrical resistance

Transendothelial electrical resistance (TEER) measured changes in monolayer integrity. To evaluate our model, we established an all-human in vitro BBB model with an EC base. Two common EC lines were used to establish the integrity of endothelial monolayers as a basis for our BBB model.

**Figure 3:**
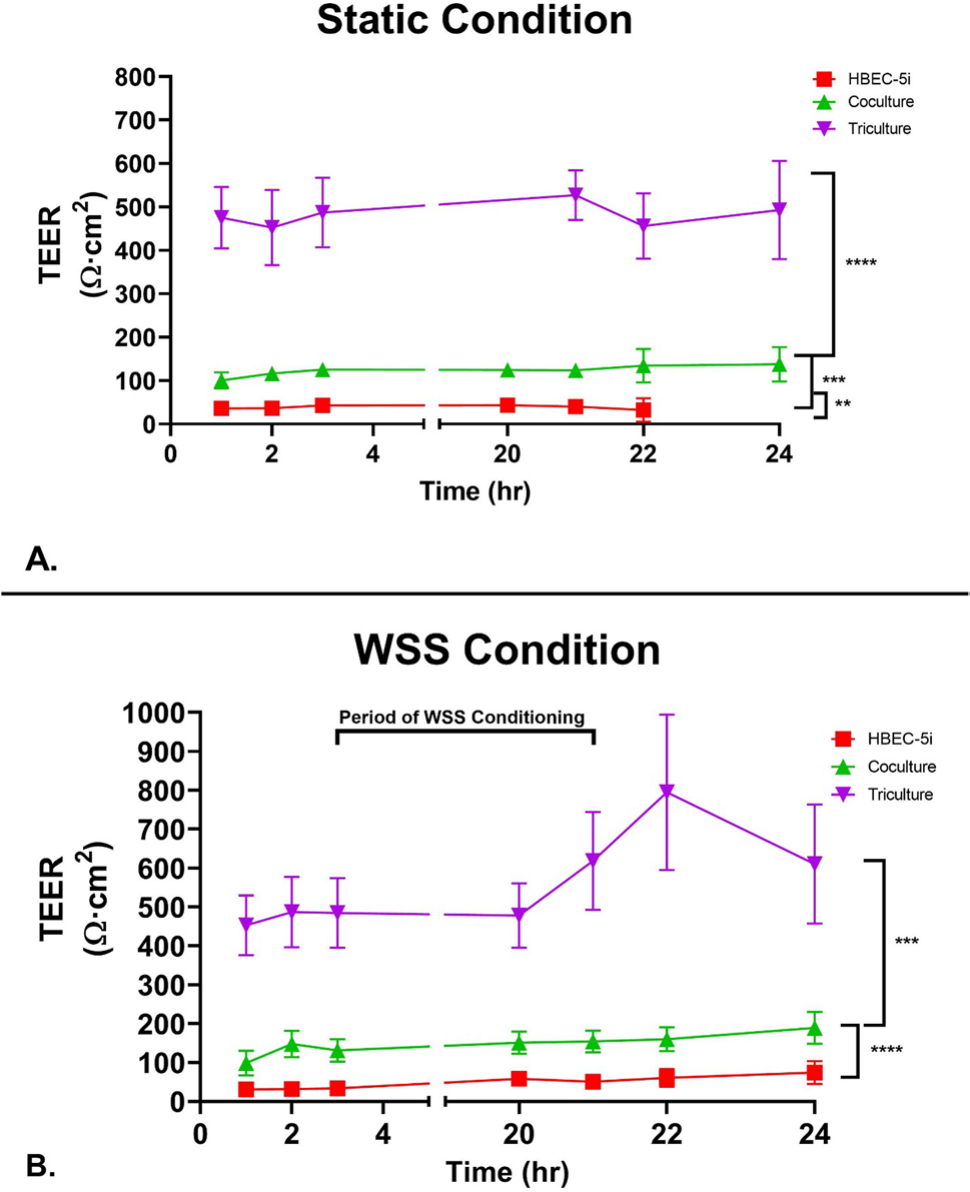
TEER measured following growth under static and dynamic conditions on Matrigel. (**A**) Confluent cultures consisting of HBEC-5i (red square), co-cultured HBEC-5i and primary human astrocytes (green triangle), and tricultured HBEC-5i, primary human astrocytes and BM-MSCs (purple inverted triangle) were established on the upper membrane of Transwell inserts. TEER was measured for a total of 24 h after addition to the platform. Data is presented as SEM. (**B**) Shear stress was applied for 18 h after a 3 h acclimation period to the platform. Confluent cultures consisting of HBEC-5i (red square), co-cultured HBEC-5i and primary human astrocytes (green triangle), and tricultured HBEC-5i, primary human astrocytes and BM-MSCs (purple inverted triangle) were established on the upper membrane of Transwell inserts and allowed to acclimatize to the system for 3 h. We then initiated WSS (4 dyne) for 18 h (t = 3 h until t = 20 h) followed by additional TEER measurements (t = 20–24 h.) Data is presented as SD. *P < 0.05; **P < 0.01; ***P < 0.001; ****P < 0.0001 (Ordinary One-way Anova). N = 24 (3 repeated experiments of n = 8 biological replicates).

HUVEC (SFig. 4a) and HBEC-5i (Fig. 3a) cultures were established on Matrigel-coated Transwells and monitored by TEER in static conditions^36–38^. Average TEER values were 14.4 ± 7.3 and 34.3 ± 10.2 Ωcm^2^ for HUVEC and HBEC-5i, respectively after subtraction from Matrigel-coated blank Transwells (SFig. 3a). To assess the glial cell response, we incorporated PHAs into the culture. HBEC-5i cells were grown with PHAs on the apical side of the Transwell to be in direct contact. In these contact coculture experiments, average TEER was significantly higher than either HBEC-5i or HUVEC in monoculture (124.6 ± 23.8 Ωcm^2^) under static conditions (Fig. 3A). To further evaluate our platform, we incorporated bone-marrow mesenchymal cell to serve as pericytes in a tri-culture model. In these contact tri-culture experiments, average TEER was significantly higher than the co-culture (474.2 ± 79.8 Ωcm^2^) under static conditions (Fig. 2). This established that our platform was capable of inducing barrier function and our independently validated software able to accurately measure responses in barrier tightness from influences with other cell types.

We next evaluated the impact of shear stress on TEER (Fig. 3B) in our model. Each endothelial construct was acclimatized in the chamber for 3 h and then exposed to WSS for 18 h (Fig. 3B). At the 21 h timepoint, shear stress was terminated and returned to the static condition. In our contact co-culture model, we measured a significantly higher TEER, 224.1 ± 62.3 Ωcm^2^, compared to monoculture. The effects of WSS on the contact tri-culture demonstrated the largest increase in TEER (714.6 ± 112.3 Ωcm^2^). The effects of WSS on the cultures supports the idea that physical force increases the integrity of endothelial barriers.

### Effects of proinflammatory mediators on TEER

**Figure 4:**
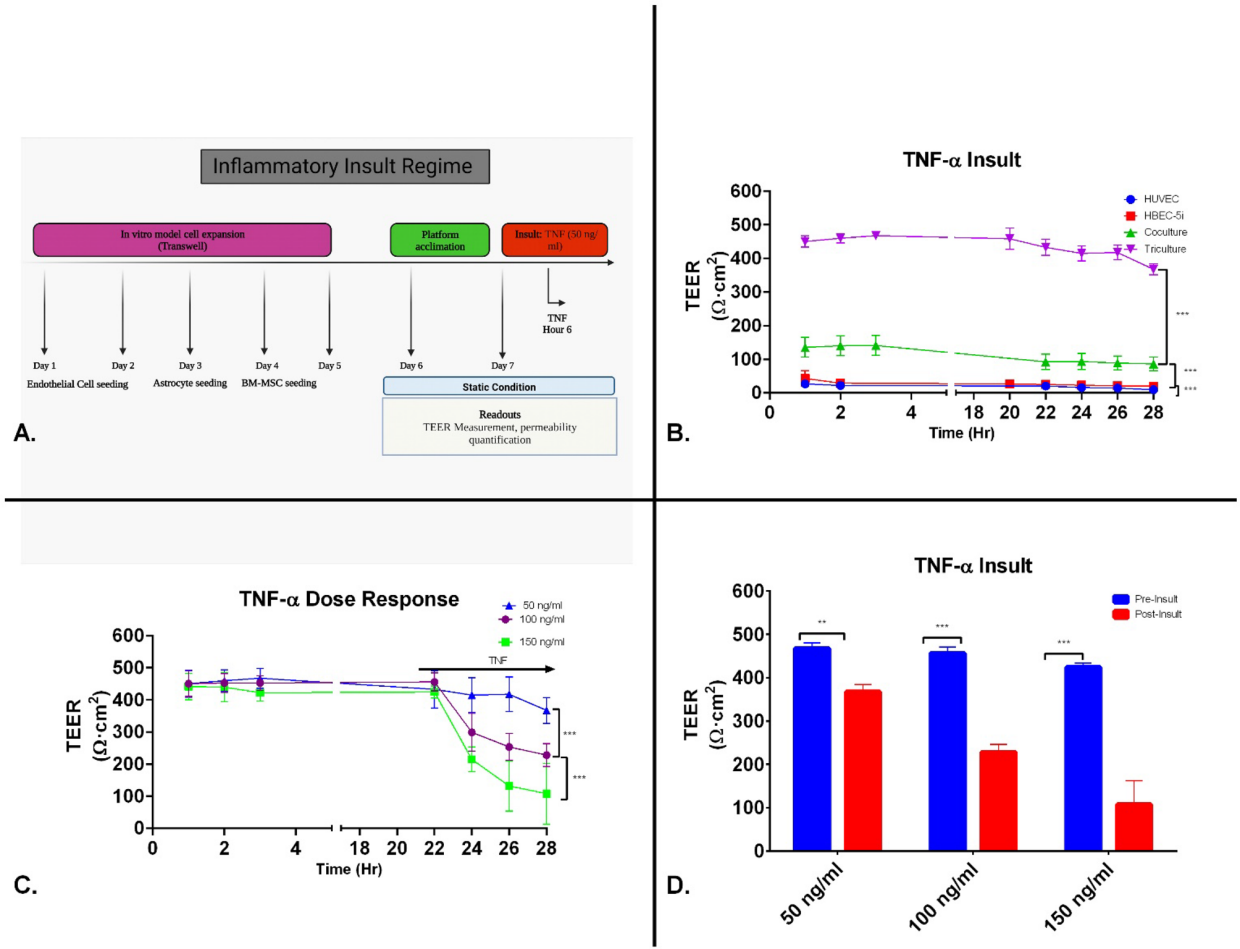
TNF-α induced changes in barrier properties of endothelial cell constructs. (**A**) Experimental timeline of inflammatory insult involving the dynamic perform. (**B**) HBEC-5is, contact coculture (HBEC-5i and primary human astrocytes) and contact tricultures (HBEC-5i endothelial cells, primary human astrocytes, and BM-MSCs) construct were grown to confluence on Matrigel coated Transwell filter inserts and added to platform. All endothelial cell constructs were allowed to acclimate in platform static flow condition for 22 h. Following TNF-α (50 ng/ml) treatment at the 24 h timepoint, TEER measurements were performed every 2 h for a total of 6 h. Data is presented as SD. (**C**) Contact triculture constructs were allowed to acclimate in platform static flow condition for 22 h. Contact tricultures were given increasing doses of TNF. TEER measurements were performed every 2 h for a total of 6 h. Data is presented as SEM. (**D**) Pre-insult corresponds to 3 h after inserts were under low flow conditions. Post-insult corresponds to 6 h after TNF exposure (Hr 28). Data is presented as SD. *P < 0.05; **P < 0.01; ***P < 0.001; ****P < 0.0001 (Two-way Anova). N = 24 (3 repeated experiments of n = 8 biological replicates).

Previous studies have demonstrated that TNF-α treatment induces an increase in paracellular permeability and/or decrease in TEER^33,34,39^. To evaluate this proinflammatory cytokine’s disruption on barrier integrity, we measured TEER after TNF-α insult. TNF-α, at a concentration of 50 ng/ml, caused an immediate but small decrease in TEER in HUVECs 2 h post-administration followed by a steady decline (average (9.2 ± 2.3 Ωcm^2^) (Fig. 4A). These early results demonstrated that our platform was able to positively respond and measure changes TEER after an inflammatory insult.

We also evaluated TEER after TNF-α insult in HBEC-5i cells. A monolayer of HBEC-5i cells responded to TNF-α with an initial decrease in TEER 2 h after administration. The contact co-culture model with astrocytes responded to TNF-α with a transient increase, then, decrease in TEER (86.1 ± 39.37 Ωcm^2^). In our contact triculture model, TNF-α decreased TEER to 367.4 ± 40.4 Ωcm^2^.

After the initial TNF studies with the contact tricultures model, we performed a dose–response to TNF-α above the 50 ng/ml dose described in the previous studies (100 and 150 ng/ml) (Fig. 4B). 100 ng/ml TNF-α decreased tricultures TEER to 228.0 ± 35.9 Ωcm^2^ while 150 ng/ml decreased TEER to 111.7 ± 24.8 Ωcm^2^ (Fig. 4C–D). Visual conformation of this response is demonstrated in SFig. 2.

Previous investigations have shown that cytochalasin (cD) negatively effects tight junction stability and could mimic the mechanical injury that occurs during TBI^40–42^. To evaluate TJ stability in our model, we used HUVEC monolayers treated with cD and measured TEER. We found that cD at a concentration of 2.5 µg/ml caused a significant decrease in TEER in baseline measurements of HUVEC cell line (6.8 ± 2.7 Ωcm^2^), consistent with previous results^43^. We also observed a significant decrease in the TEER of HBEC-5i monolayers (average of 18.4 ± 6.3 Ωcm^2^), the TEER of our co-culture models (96.9 ± 31.3 Ωcm^2^) and the TEER of our tri-culture model (361.5 ± 131.3 Ωcm^2^) (SFig. 5).

### Effects on TEER after OGD treatment and reperfusion

**Figure 5:**
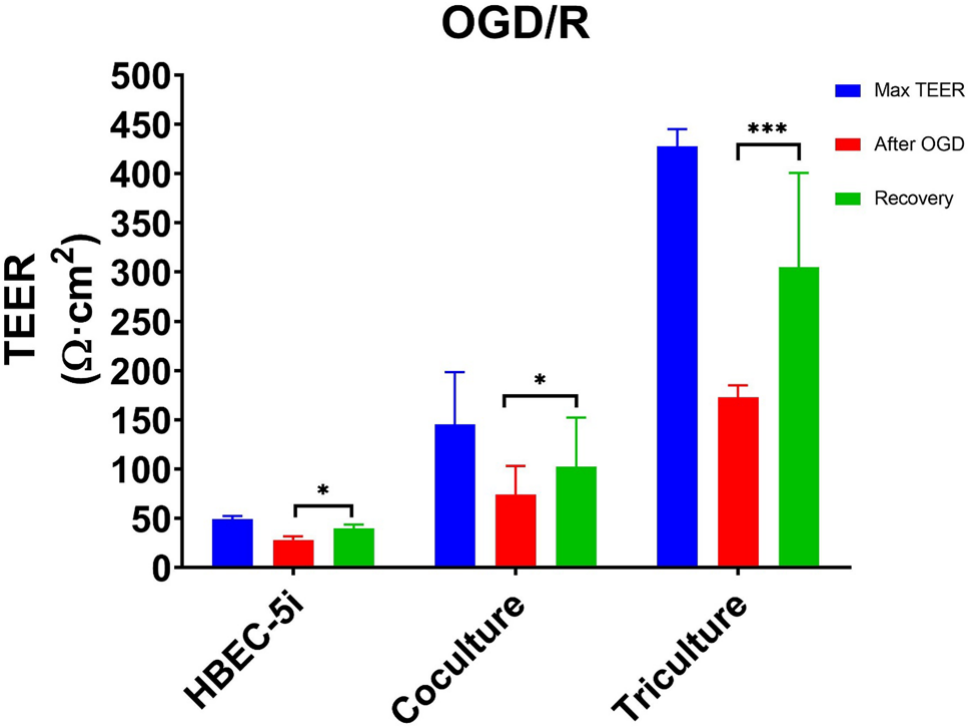
Effects of an oxygen and glucose deprived environment on TEER. Confluent cultures consisting of HBEC-5i, co-cultured HBEC-5i and primary human astrocytes, and tricultured HBEC-5i, primary human astrocytes and BM-MSCs were established on the upper membrane of Transwell inserts and allowed to acclimatize to the system for 20 h (Max TEER). After 4 h of exposure to an OGD environment (After OGD), monolayers had developed a significant decrease in their optimal resistance. Monolayers were reperfused with original growth media and allowed to recover for 24 h (recovery). Data is presented as SD. *P < 0.05; **P < 0.01; ***P < 0.001; ****P < 0.0001 (Two-way Anova). N = 24 (3 repeated experiment of n = 8 biological replicates).

The effects of oxygen–glucose deprivation (OGD) and reperfusion were evaluated in the platform (Fig. 5). TEER was measured at baseline, after 4 h of OGD, and after 24 h of reperfusion. The max TEER of the HUVEC, HBEC-5i, contact coculture and contact triculture models were 33.0 ± 5.4, 42.1 ± 6.4, 116.4 ± 38.8 and 466.4 ± 93.8 Ωcm^2^, respectively, measured during baseline. TEER decreased significantly after 4 h of OGD (13.1 ± 2, 35.3 ± 16.1, 99.7 ± 79.1, 163.6 ± 38.8 Ωcm^2^) as compared to the peak resistance. After reperfusion, the TEER recovered to 23.7 ± 0.3 Ωcm^2^ in HUVEC monolayers, 35.3 ± 16.1 Ωcm^2^ in HBEC-5i monolayers, 129.2 ± 48.9 Ωcm^2^ in the contact coculture model, and 329.2 ± 78.9 Ωcm^2^ in the contact triculture model. These results demonstrate that our dynamic platform detects changes in TEER in response to metabolic insults.

### Evaluation of paracellular permeability through the modular platform

**Figure 6:**
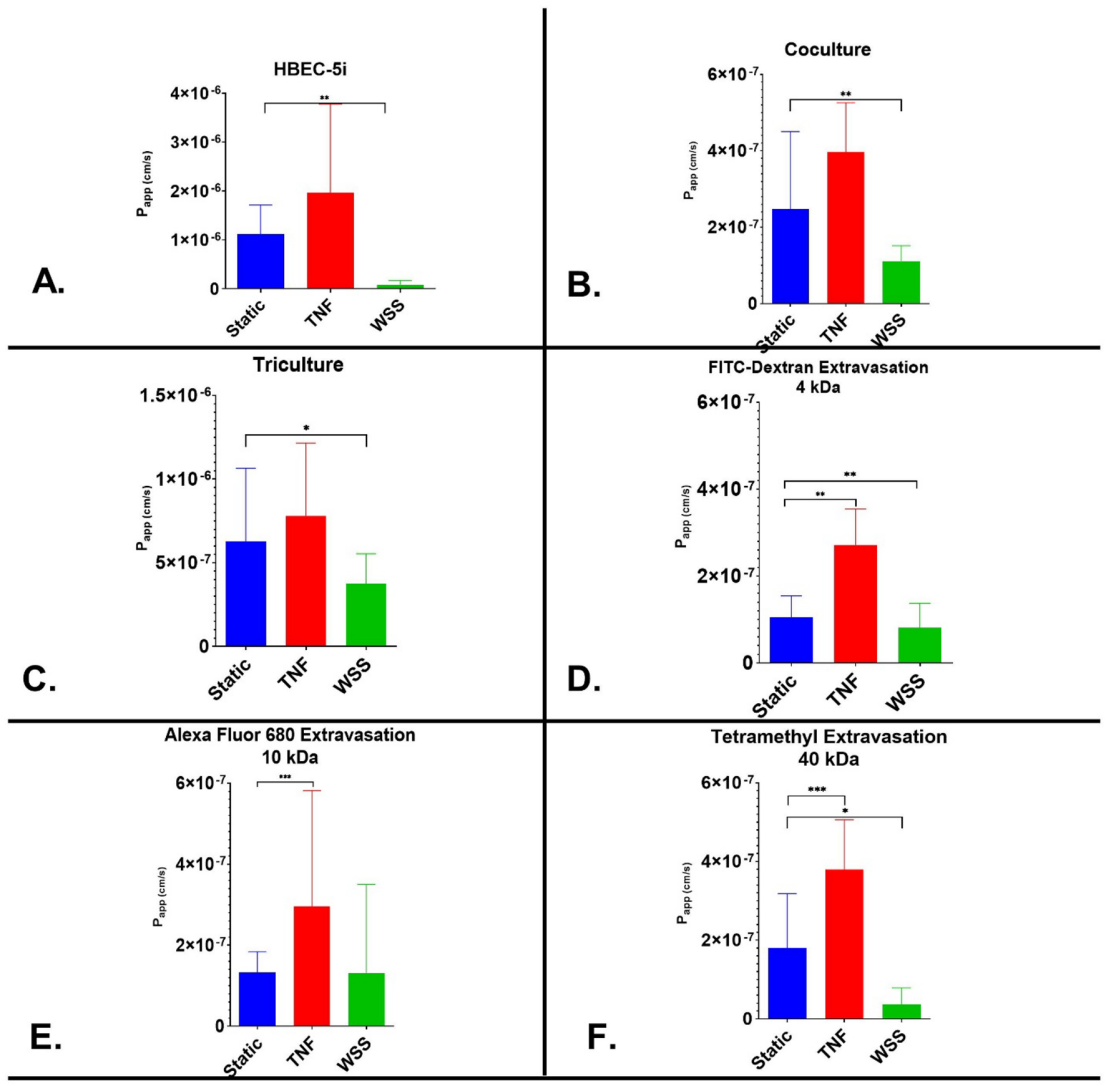
Mean *P*_app_ values of variable sized tracers in endothelial cell monolayers constructs. The permeability coefficient of 15 kDa FITC-Dextran after static condition, the application of WSS, and treatment with proinflammatory cytokine TNF-α in (**A**) HBEC-5i; (**B**) Direct contact coculture; and (**C**) Contact triculture. The permeability coefficient of (**D**). 4kda FITC-Dextran (**E**) 10 kDa Alexa Fluor 680 and (**F**) 40 kDa Tetramethyl-Dextran were further evaluated in the contact triculture model. Data is presented as SD. *P < 0.05; **P < 0.01; ***P < 0.001; ****P < 0.0001 (Ordinary One-way Anova). N = 24 (3 repeated experiment of n = 8 biological replicates).

To further validate our platform, we performed permeability assays to assess paracellular transport in our dynamic platform (Fig. 6) by measuring the transport of a 15 kDa FITC-labeled dextran molecule from the luminal to abluminal side (calculated as the *P*_app_). In HUVECs, the P_app_ was determined to be 8.18 × 10^–7^ cm s^−1^ (SFig. 6). TNF-α did not alter the permeability of HUVEC monolayers. The application of WSS on HUVECs produced a significantly lower permeability coefficient (*P* < 0.05) than observed under static conditions. For the HBEC-5i cell line, the *P*_app_ to 15 kDa FITC-dextran was 1.1 × 10^–6^ cm s^−1^ under static conditions. TNF-α did not produce a significant effect. The application of WSS on the HBEC-5i monolayer construct produced a significantly lower permeability coefficient of 8.4 × 10^–8^ cm s^−1^. The coculture construct produced a *P*_app_ value of 6.3 × 10^–7^ cm s^−1^ under static conditions. TNF-α exposure did not result in a significant increase in15 kDa FITC-dextran permeability (*P* > 0.2012). The application of WSS did cause a significant decrease in the *P*_app_ (3.8 × 10^–7^ cm s^−1^). In the triculture model, the *P*_app_ was determined to be 2.5 × 10^–7^ cm s^−1^ under static conditions. The application of WSS on HUVECs produced a significantly lower permeability coefficient (*p* < 0.05) than observed under static conditions. The addition of TNF-a did not significantly change permeability in triculture construct, while application of WSS significantly reduced permeability in HBEC-5i and HUVEC monocultures. This data supports that WSS is important for establishing a relevant resistance barrier and inducing changes in barrier integrity. To further evaluate the microvascular permeability of the contact triculture, variable sized molecules (4, 10, & 40 kDa) were evaluated under static condition, TNF inflammatory insult (100 ng/ml) and WSS. Inflammatory insults caused increased in permeability using 4 kDa (2.5 × 10^–7^ cm s^−1^) (Fig. 6D), 10 kDa (2.7 × 10^–7^ cm s^−1^) (Fig. 6E), and 40 kDa (3.7 × 10^–7^ cm s^−1^) (Fig. 6F). In comparison to the static condition, WSS produced a significantly lower permeability coefficient (*p* < 0.05) with each size of the molecular tracers (4 kDa − 7.9 × 10^–8^ cm s^−1^ (Fig. 6D); 10 kDa − 4.3 × 10^–8^ cm s^−1^ (Fig. 6E); & 40 kDa − 3.7 × 10^–8^ cm s^−1^) (Fig. 6F).

### Triculture hydraulic conductivity

**Figure 7:**
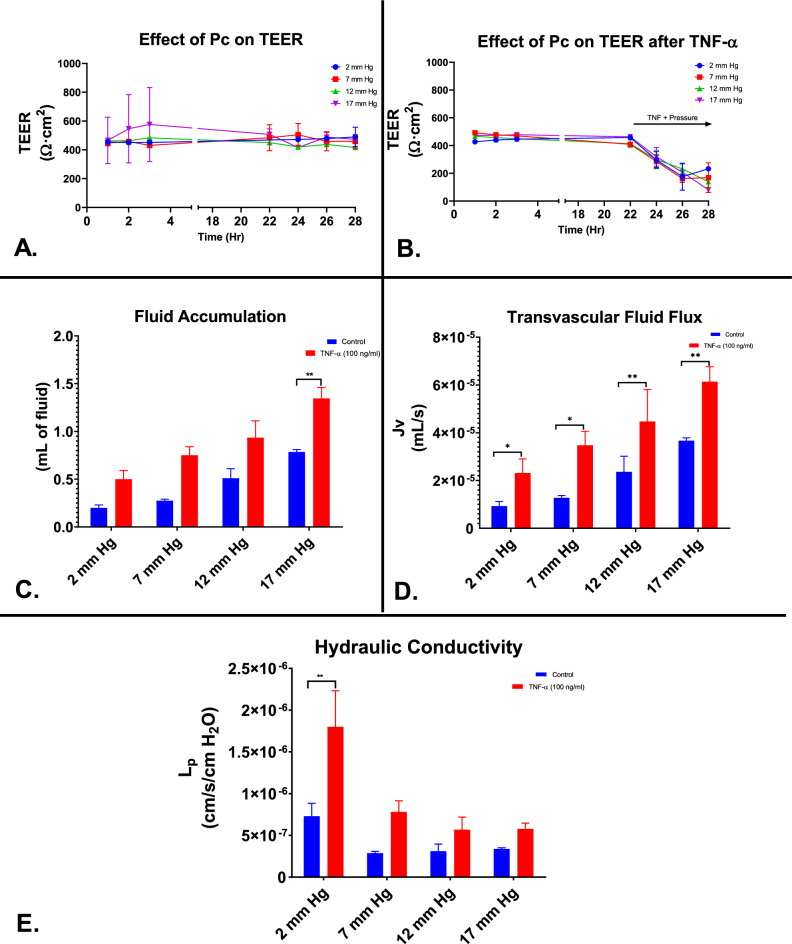
Pressure effects and hydraulic conductivity of the contact triculture in vitro. (**A**–**B**) Contact triculture constructs were allowed to acclimate in platform static flow condition for 22 h. Following TNF-α and pressure induction treatment after the 22 h timepoint, TEER measurements were performed every 2 h for a total of 6 h. Data is presented as SEM. (**C**-E) Hydraulic conductivity experiments were performed as described in the "Materials and Methods" sections. Fluid accumulation was collected for a total of 6 h under increasing pressure (2–17 mm Hg). Data is presented as SD. *P < 0.05; **P < 0.01; ***P < 0.001; ****P < 0.0001 (Two-way Anova).

To evaluate the effects of hydrostatic pressure and TNF-α (Fig. 7A–B) in the triculture system, TEER was assessed over variable pressures ranging from 2 to 17 mm Hg. Average TEER vales at 2 mmHg hydrostatic pressure (Fig. 7A) was 466.0 ± 35.2 Ωcm^2^, 463.2 ± 61.6 Ωcm^2^ at 7 mm Hg, 447.8 ± 61.7 Ωcm^2^ at 12 mm Hg, and 502.4 ± 135.8 Ωcm^2^ at 17 mm Hg. Upon inflammatory treatment with TNF-α (100 ng/ml) (Fig. 7B), the average TEER values at 2 mmHg hydrostatic pressure was 353.3 ± 112.7 Ωcm^2^, 328.8 ± 147.2 Ωcm^2^ at 7 mm Hg, 340.8 ± 129.8 Ωcm^2^ at 12 mm Hg, and 356.2 ± 155.8 Ωcm^2^ at17 mm Hg The volume of fluid accumulated in the reservoirs after pressure induction and TNF-α exposure were obtained after 6 h. The mean amount of fluid collected from control contact tricultures after pressure induction at 2 mm Hg was 0.2 mL, 0.275 mL at 7 mm Hg, 0.5 mL at 12 mm Hg, and 0.8 mL at 17 mm Hg (Fig. 7C).The mean amount of fluid collected from TNF-α exposed contact tricultures after pressure induction at 2 mm Hg was determined to be 0.5 mL, 0.8 mL at 7 mm Hg, 0.9 mL at 12 mm Hg, and 1.3 mL at 17 mm Hg. The mean hydraulic conductivity of contact tricultures at 2 mm Hg was determined to be 8.4 × 10^−7^, 3.0 × 10^−7^ at 7 mm Hg, 2.5 × 10^−7^ at 12 mm Hg, and 3.3 × 10^−7^ at 17 mm Hg. The mean hydraulic conductivity of contact tricultures exposed to 6 h TNF-α (100 ng/ml) at 2 mm Hg was determined to be 1.8 × 10^−6^, 7.8 × 10^−7^ at 7 mm Hg, 5.7 × 10^−7^ at 12 mm Hg, and 5.8 × 10^−7^ at 17 mm Hg (Fig. 7E). There was a significant difference in hydraulic conductivity between control contact tricultures (*p* < 0.01) and TNF-α exposed contact tricultures.

## Discussion

This study describes the development of a novel BBB platform for interrogating TBI’s effect on the BBB using a system that can simulate physiological forces to better understand clinical conditions. A number of promising preclinical therapeutics from simplified experimental models have failed to perform when translated to real world conditions during clinical trials. Accurate i*n vitro* modeling of the BBB in trauma is complicated by the complex pathophysiology of TBI across multiple tissues and systems incorporating inflammatory signaling, mechanical forces, and metabolic stress. Appropriately testing therapeutic paradigms requires a model that can recapitulate capillary hemodynamics, intracranial hydraulics, and BBB’s unique cellular relationships. Considering this, we designed a modular platform that allows the imposition of pressure, the direct measurement of transvascular fluid flux, the application of shear stress and the compartmentalization of distinct cell populations.

Given its ease of use and adaptability, a great deal of biological barrier research has utilized the Transwell model and a vast body of literature exists for benchmarking our innovative platform^4,26,44–48^. A key constraint of our system was working with existing Transwell culture inserts that prevented us from being able to modify the geometry of the wells, such as using perfect cylinders to simplify calculations and improve the junctional tightness to prevent leaks from forming under pressure. These disadvantages were outweighed by their prior characterization, utility, reproducibility, and commercial availability of the existing manufactured Transwell insert systems^49^. Our modular BBB platform improves on these shortcomings by incorporating dynamic features, such as wall shear stress (WSS) and flow rate-independent pressure. There are limited BBB models that possess the ability to modulate pressure in their systems. However, our platform retains the Transwell model’s utility for high-throughput experimentation and multi-modal barrier assessment. The platform’s components are conventionally fabricated, and cellular constructs can be developed at scale outside of the device following well-established protocols.

A number of previous efforts have been made to use Transwell systems to model endothelial barrier dynamics including the BBB and NVU. The complexity of these systems has varied, with some products now even featuring multi-lineage mixed cell components (alphabioregen). Among several notable examples is a multicellular system developed by Janigro and Cucullo and colleagues^18,50–52^. In a set of elegant studies, the investigators used multicellular cultures in both static and dynamic fluid systems that addressed the effects of inflammatory cytokines and metabolic stress on the integrity of their BBB model assessed using TEER and a number of biochemical assays. Our study attempts to iterate on this previous work by increasing the throughput in order to better evaluate putative therapeutic interventions and using a sealed system to modulate pressure independent of fluid flow to better model the hydrodynamic forces in clinical ICP.

There are a number of other emerging in vitro models that simulate elements of the BBB and NVU, such as lab-on-a-chip barriers, tissue-engineered models, and the DIV-BBB models^15,18,51,53,54^. While these complex models represent a significant advancement in current in vitro BBB models, system setup is quite complex, requiring significantly greater time, resources, and technical skills than conventional platforms. These constraining factors, unfortunately, limit the adoption and further development of these models for high-throughput translational research. Finally, most evolving models lack features for manipulating fluid dynamics in a manner relevant to TBI.

To validate our platform in comparison to current in vitro models, we measured the TEER and selective permeability of four cellular constructs under various experimental conditions after we established the expression of cell surface specific markers (Fig. 2). This initial characterization of surface markers was important to establish the interactions of the individual components of the BBB. As cells are now aligned in a 3-D configuration based off of Matrigel-coating, we demonstrate the complexity of the interactions used in our platform as well as the difficulty in visualizing the expected orientation and distribution of tight junction-associated proteins in our immunostained transwells. We specifically note that our ZO-1 and claudin-5 staining are not localized clearly to the outer membranes of the endothelial cells to form a monolayer barrier amidst the 3 dimensional culture system. This lack of cellular and protein organization makes it difficult to identify visually whether the cells are forming tight junctions or merely expressing tight junction proteins. While the tri-culture functions and responds to stimuli as expected and previously reported, future studies of our triculture system are required to definitively relate changes in our model’s barrier integrity to changes in tight junctions.

Historically, TEER has served as the benchmark standard for in vitro BBB characterization^56^. The accuracy of this measurement is sometimes limited in tissue engineered BBB models due to electrode placement or the use of ohmmeters rather than impedance spectroscopy. Measuring via impedance spectroscopy provides a more accurate representation of actual TEER and can provide additional information about the capacitance of the cell layer^29^. We note the relationship between TEER and permeability is non-linear and highly dependent on the biochemistry of the endothelial cell type^57,58^.

To obtain more physiologically relevant barrier integrity, we incorporated tunable fluid flow into our experimental design. WSS has been extensively reported to play an important role in the morphology and functionality of brain microvascular endothelial cells, the control of transport processes in the NVU, and the expression of critical protein complexes for the BBB phenotype. The average shear stress within the arterial circulation is 4–30 dyne cm^2^ and 1–4 dyne cm^2^ in the venous circulation^50^. In vitro models that have imposed WSS have demonstrated lower permeability coefficients to molecular tracers, indicating the formation of a stable BBB phenotype, and enhanced expression of BBB specific markers^59,60^.

To more rigorously validate our novel platform, we utilized a combination of EC lines (HUVEC and HBECi-5), human astrocytes or primary bone marrow derived-mesenchymal stem cells. HUVECs were chosen as the first endothelial cell line because of their restrictive barrier properties. HUVECs have exhibited at low passages (3–7) relative high TEER and low solute permeability. The HBEC-5i line was chosen over alternatives such as the hCMEC/d3 cell line. HBEC-5i have demonstrated potential for in vitro permeability screening. This immortalized cell line have been observed to express critical tight junction-associated proteins as well as provide high TEER and low permeability comparable to other immortalized BMECs. However, other studies and our own experience found that HBEC used at passage 3–5, did not have the typical cellular localization of tight junction-associate proteins^55^. It has been reported that immortalized cells tend to exhibit a lower TEER value in comparison to primary or stem cell derived cell lines^16^. Given this potential for low TEER, we co-cultured primary astrocytes and/or BM-MSCs with the HBEC-5i cell line to improve the BBB phenotype^16,44,61–63^. After initial expansions following the supplier’s recommendation, each cell construct was cultured on Matrigel coated Transwell systems for up to five days.

Our use of HUVECs to optimize our equipment and culture systems is similar to many other groups^64^. However, it is important to note that HUVECs are not a suitable surrogate for brain microvascular endothelial cells for a number of reasons, including differences in immune cell interactions^65^, barrier function^66^, and can exhibit wide variability based on source^67^, and time in culture^55^. For these reasons, the direct comparison of our data utilizing HUVECs to HBECs has limited use.

HUVEC monolayers exposed to WSS reached a peak TEER of 61 Ω cm^2^ compared to that of static, 21 Ω cm^2^ (SFig. 4). We measured a peak of TEER of 74 Ω cm^2^ in HBEC-5i endothelia exposed to WSS, as compared to 36 Ω cm^2^ under static samples (Fig. 3b). The observed TEER of static HBEC-5i monolayers is similar to those reported by Puech et al. (36 Ω cm^2^) and values found in a review of in vitro models using immortalized human brain endothelial cells in monoculture (40 Ω cm^2^). Our reported *P*_app_ to fluorescein before shear stress conditioning (1.1 × 10^–7^ cm s^−1^) and after (8.4 × 10^–8^ cm s^−1^) was lower than reported in other studies using immortalized human brain endothelial cells^68^. The 2–threefold increase in membrane resistance strongly suggests the positive impact of shear stress on barrier function and resembles the results reported in literature focusing on dynamic systems. Using their BBB microfluidic platform, Griep et al. reported a threefold TEER increase after exposing hCMEC/D3 monolayers to 5.8 dyne/cm^2^, a slightly higher shear stress than we imposed. This phenomenon has been attributed to cytoskeletal rearrangements, tight junction formation, and morphological changes incited by WSS^26,53^.

In astrocyte coculture, we noted a 263% increase in peak TEER under static condition compared to mono-cultures (Fig. 3a). The peak TEER after WSS conditioning, 210 Ω cm^2^, is higher than reported with indirect and direct co-culture constructs involving immortalized EC and astrocytes^44^. For example, hCMEC/D3 cultured with astrocytes in Transwell^Ⓡ^ inserts attained a TEER of approximately 70 Ω cm^2^ after 9 days of static culture. It is also greater than the observed TEER (39.8 cm^2^) of endothelial cells cultured in astrocyte conditioned medium^68^. Furthermore, our static co-culture was characterized by a lower permeability to FITC-dextran than endothelial cells cultured in astrocyte conditioned medium (*P*_app_ = 7.7 × 10^–7^ cm*s^−1^ versus 3.6 × 10^–6^ cm*s^−1^) (Puech et al. 2018). The increases in membrane resistance after the addition of astrocytes is most likely the result of cell-to-cell communication via soluble growth factors. Evidence suggests mechanical cues play a role in exciting astrocyte signaling and their regulation of endothelial cells^15^. These results confirm that our platform facilitates endothelial cell responses to stimuli from glial cells, similar to the in vivo endothelium.

The addition of BM-MSCs to the cell construct were to further evaluate constitutive members of the BBB. Work over the last few years have demonstrated a strong similarity between MSC and pericytes^69^. MSCs and pericytes express many of the same phenotypic markers as well as demonstrate some functional equivalence to the diverse mechanisms in which homeostasis is maintained in their respective cellular environments as noted in Stable 1^70,71^. The incorporation of these mural-like cells noted a 275% increase in peak TEER under static condition compared to the coculture. The peak TEER after WSS conditioning, 713 Ω cm^2^, a TEER significantly higher than reported previously with primary pericytes^72^. Furthermore, our static triculture was characterized by a lower permeability to 15 kDa FITC-dextran than our co-culture (*P*_app_ = 6.3 × 10^−7^ cm*s^−1^ versus 1.1 × 10^−6^ cm*s^−1^). The increases in membrane resistance after the addition of BM-MSCs is most likely the result of cell-to-cell communication via soluble growth factors. These results suggest in a well-established BBB model, MSCs play similar roles to pericytes in cell culture. After validation, we determined that the direct contact triculture model would be used for future studies characterizing cellular responses within the BBB. The direct contact triculture is more physiologically relevant to the in vivo BBB that is observed in the NVU and does not require manipulation of the Transwell system and potentially is more amenable to automation for higher capacity throughput screening.

After initial characterization, we evaluated the effect of biochemical insults in our platform. Clinical and preclinical studies have demonstrated that within minutes of a traumatic impact, a robust inflammatory response is elicited in the injured brain^14,73–75^. This response is characterized by the secretion of proinflammatory cytokines, such as TNF-α, by microglia, astrocytes, and other members of the NVU^34,76,77^. To experimentally mimic the molecular events underlying cerebrovascular barrier breakdown post-TBI, the administration of TNF-α is commonly used to induce an increase in endothelium permeability. In this study, we demonstrated that TNF-α (50 ng/ml) negatively effects EC microvascular integrity (Fig. 4a). This supraphysiological dose is outside of the typical dose observed in vivo but provided an opportunity to measure TEER dynamic responses from the platform. The microvascular integrity effects observed in this study are potentially mediated through cytoskeletal rearrangements of ZO-1 or VE-cadherin. A study in primary cultures of retinal endothelial cells demonstrated that TNF-α induces changes in protein levels of ZO-1 and claudin-5 as early as 6 h after treatment, similar to our observations^78^. Studies involving peripheral endothelial cells such as the HUVEC cell line observe changes in TEER through the second mechanism. These studies report a focal loss of VE-cadherin–mediated intercellular adhesion and degradation of the tight junction protein occludin in areas of endothelial barrier breakdown after TNF-α in similar concentrations^33,34^.

Due to its fast action, we also utilized cytochalasin-D (cD) to assess the endothelial cell response to biochemical insults using our platform. cD is a specific inhibitor of actin polymerization^33^. Multiple studies have demonstrated that actin cytoskeleton depolymerization plays an important role in mechanical stress in TBI during neuroinflammation, which often leads to apoptosis^79^ The response of cD was rapid in each construct with decreases in membrane resistance observed within 2 h of exposure. cD decreased the HUVEC monolayer TEER by over 87% and HBEC-5i monolayer TEER by 43%. The contact co-culture was more resistant to the cD insult and decreased by 28% (SFig. 5). The results are similar to those reported in the literature for endothelial cell monolayers^76^.

Given the clinical importance of hypoxia-reperfusion injury to outcomes in TBI, we considered the OGD/R assay critical to the platform’s validation^80,81^. The OGD/R model is one of the most common experimental protocols used to mimic ischemic stroke and TBI *in vitro*^72,82,83^. To execute the assay, our platform was sealed to the external environment and medium was replaced with oxygen depleted, glucose free media for 4 h. After 4 h, the capillary compartment was re-perfused with fresh medium and opened to the incubator environment for 24 h. The 4 h metabolic insult induced significant decreases in TEER for each of the endothelial monolayers. In the reperfusion after metabolic deprivation, we observed increased TEER in our contact coculture and HUVEC constructs (SFig. 7). While TEER did not return completely to baseline during the experiment in monolayer inserts, this is most likely due to changes in cell to cell connecttions^64,66,67^.

One of the major design advantages of our dynamic platform is the ability to impose variable hydrostatic pressure across cultures from the luminal to the abluminal side. This platform was designed with specialized tubing that when connected in a series increases the pressure in each chamber/insert. Together with TNF-α treatment (100 ng/ml), we assessed the mean hydraulic conductivity in our tricultured model. This TNF dose was previously determined in the dose–response trial to reduce the microvascular barrier integrity significantly. The mean hydraulic conductivity we calculated in our triculture model is comparable to other studies in the literature that have utilized a restrictive microvascular endothelial cell, while being significantly lower than published macrovascular endothelial cell lines in vitro. As expected, the mean hydraulic conductivity decreased as pressure increased in our platform (Fig. 7). The low hydraulic conductivity that is normally observed in the BBB may serve to critically maintain brain homeostasis by regulating concentrations of ions needed for cerebral blood flow and metabolism. This function is also a potential mechanism for balancing the development of cerebral edema following severe disruptions in ICP. The hydraulic conductivity assay provides a mechanism for evaluating novel therapeutics in the treatment of TBI and determining the pathways responsible for Transendothelial fluid flux across the BBB. This will potentially provide important knowledge of an essential restrictive microvascular biophysical property, in addition to TEER and solute permeability.

With this study, we validated a novel *in-vitro* platform that recapitulates significant portions of the blood–brain barrier’s physical environment and imposed physical conditions similar to post-capillary venules (WSS = 4 dyne/cm^2^, Capillary Pressure = 2–4 mmHg) via thickened media with a similar viscosity to blood (0.0345 dyne*s/cm^2^). Importantly, the parameters of WSS, interstitial pressure, and capillary pressure can easily be tuned to reflect pathological disturbances or alternative segments of vasculature.

As a dynamic model to study NVU interactions after TBI, we recognize that our model does not contain all of the elements of the NVU. For example, microglia, neurons, oligodendrocytes, etc. were not included in our system^84^^,^^85^. We restricted our initial characterization efforts to primarily use ECs (HUVEC and HBEC-5i) for simplicity and to directly compare the performance of our system with similar studies. We then introduced PHAs to demonstrate the feasibility of multi-cell culture systems and plan to eventually incorporate additional cell types. We are particularly interested in incorporating microglia, as a number of our other studies have found that these resident brain immune cells are important in TBI pathology and may be key to the therapeutic effects of a number of experimental treatments for TBI^86–89^. We also understand that our model does not include a direct impact or mechanical injury that is typically observed with in *vivo* TBI. We have included the cD as a proposed mechanism to simulate the mechanical injury observed in the BBB endothelium after TBI. It is our goal to develop/and or incorporate models of impact injury that can be observed in vitro.

In the future, we expect to recapitulate more aspects of the NVU, especially additional cellular constituents and blood as the capillary fluid. In regards to TBI, the platform invites interrogation of different capillary and interstitial pressures effect on barrier formation and resilience. As more of the TBI cascade is modelled with the high-throughput platform, we hope to gain translational insights that might guide clinical guidelines and therapeutic development.

The validity of in vitro models has largely been assessed by TEER and permeability to various molecules, with TEER measured via non-equivalent techniques^90^. In past years, models have also been criticized for their architecture, protein expression, morphology, and physiologic relevance^91^. It was necessary, especially given the cascade of events seen in neuropathology, that recapitulating the inciting events and sequelae are critical to the validity of the model and attributes to the TBI disease model. We have improved on these shortcomings by incorporating real-time monitoring with the possibility of post-hoc analysis without compromising cellular integrity as the BBB on a chip model. This design should improve the translation of our preclinical therapeutics to clinical outcomes when used to treat TBI and other neurocritical indications.

### Supplementary Information

Supplementary Figures.

## Data Availability

The datasets used and/or analyzed during the current study available from the corresponding author on reasonable request.
