# Supplementary material for: Validation and characterization of a novel blood–brain barrier platform for investigating traumatic brain injury
Source: Sci Rep. 2023 Sep 26;13:16150. doi: 10.1038/s41598-023-43214-7 (PMC10522590; doi:10.1038/s41598-023-43214-7)
Supplement: Supplementary file 1 — Supplementary Figures. [file 41598_2023_43214_MOESM1_ESM.docx]

Validation and Characterization of a Novel Blood-Brain Barrier Platform for Investigating Traumatic Brain Injury

Authors: Christopher T. Bolden, PhD^1,2*^ & Max A. Skibber, MS^1^, Scott D. Olson, PhD^1^, Miriam Zamorano Rojas, PhD^1^, Samantha Milewicz^1^, Brijesh S. Gill, MD^3^, Charles S. Cox Jr, MD^1,2,4*^


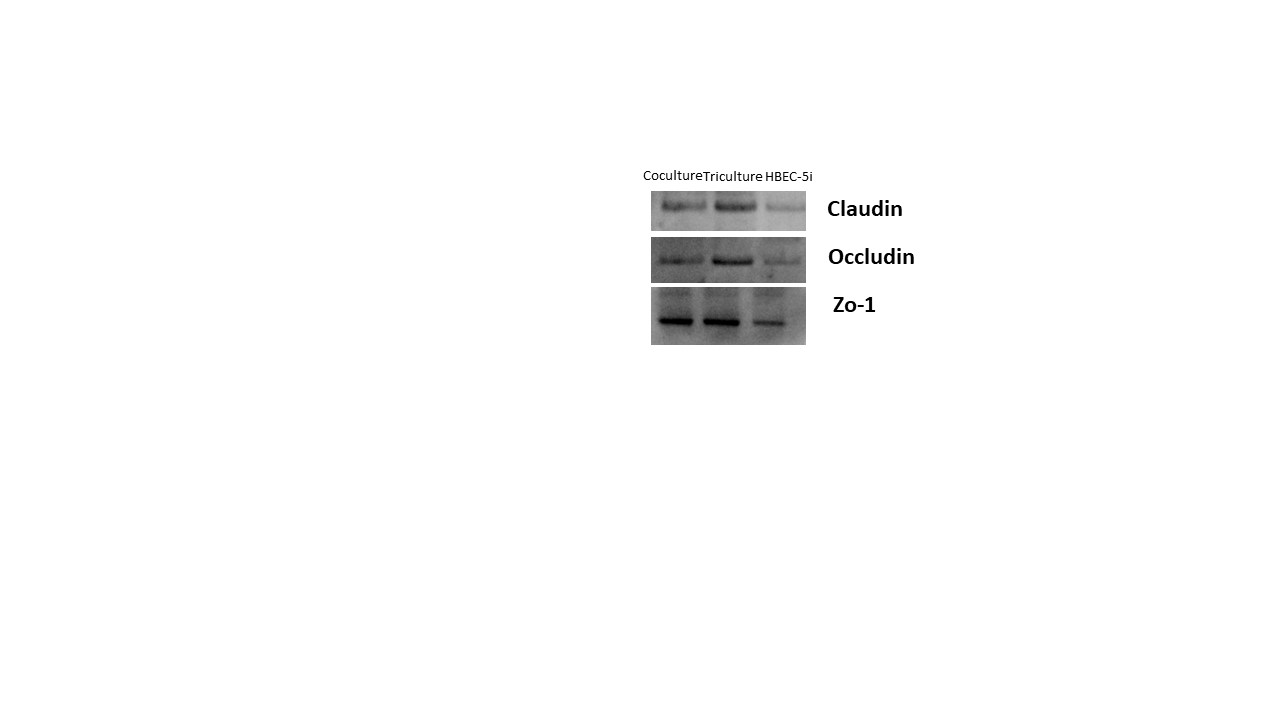


**Supplemental Figure 1A. Western Blot of Tight Junction Proteins in model construct**. Claudin-5, occludin, and zo-1 expression were evaluated in the HBEC-5i monoculture, coculture with primary human astrocytes, and contact triculture with BM-MSCs.


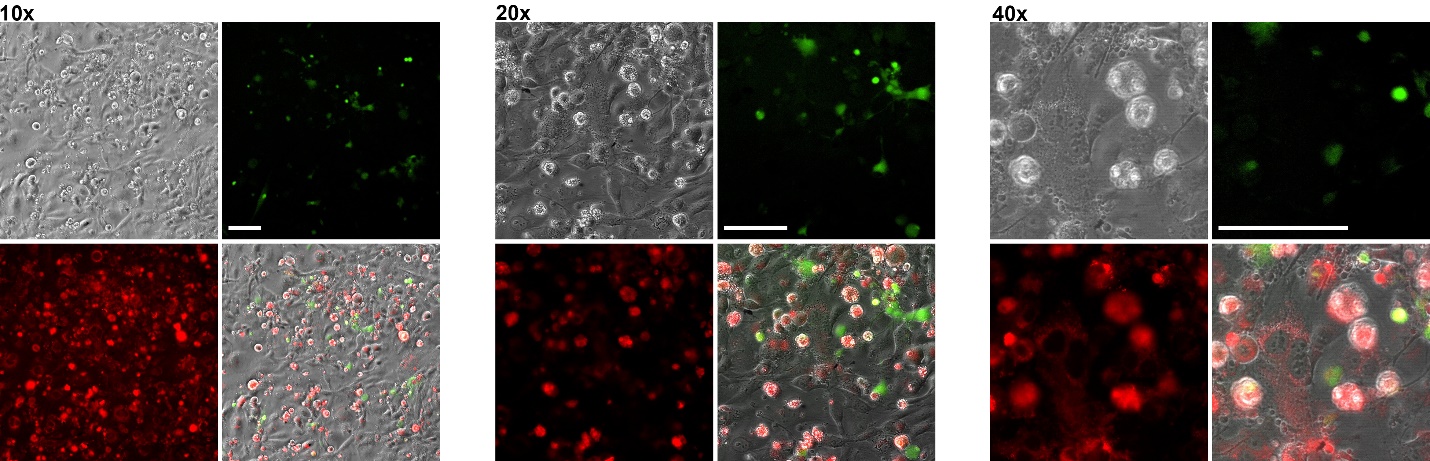


**Supplemental Figure 1B. CellTracker staining of contact triculture**. Contact triculture of HBEC-5i (unstained), primary human astrocytes (green, CMFDA), and BM-MSCs (red, CMFPTX) in Lab-tek chamber slides. Scale bar 100 µm.

**
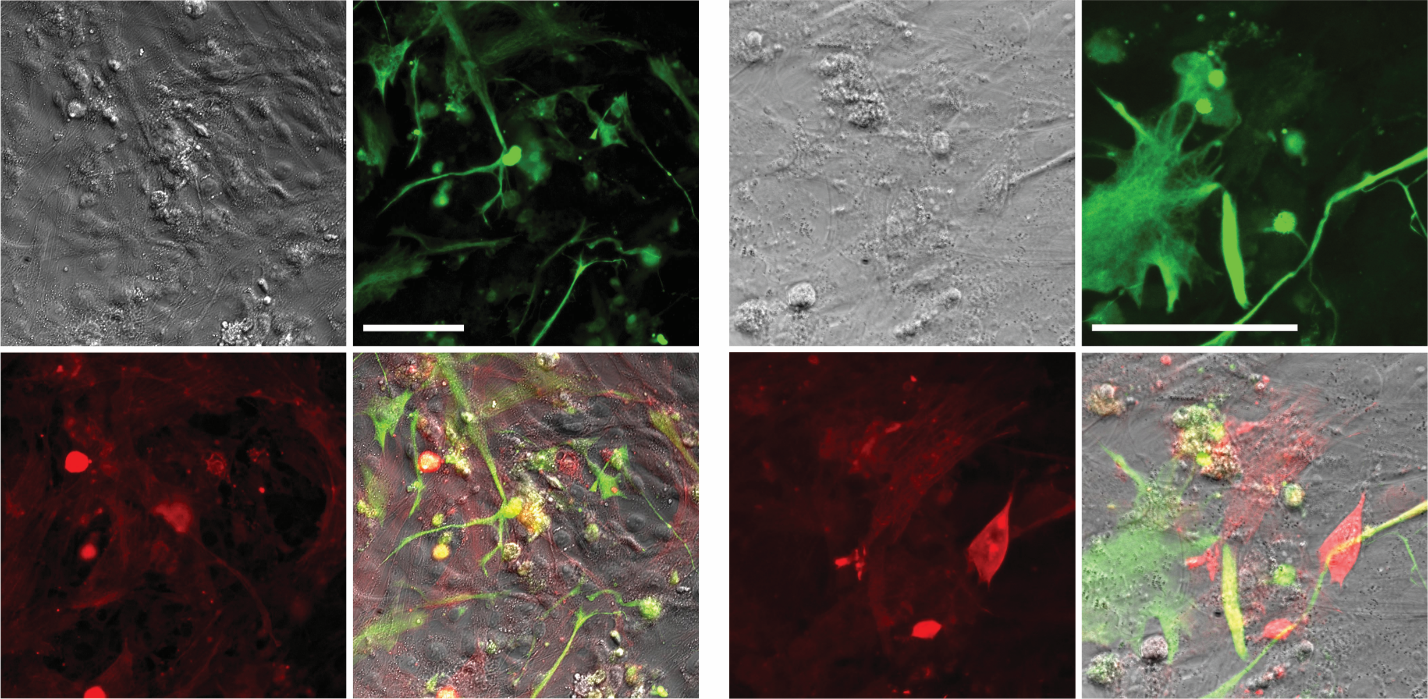
**

**Figure 1C. Immunostaining of contact triculture BBB model.** Fluorescent cell marker expression of triculture model components including HBEC-5is (Phase, Gray), primary human astrocytes (GFAP, Green), and BM-MSCs (α-smooth muscle actin, red). Scale bar 100 µm.


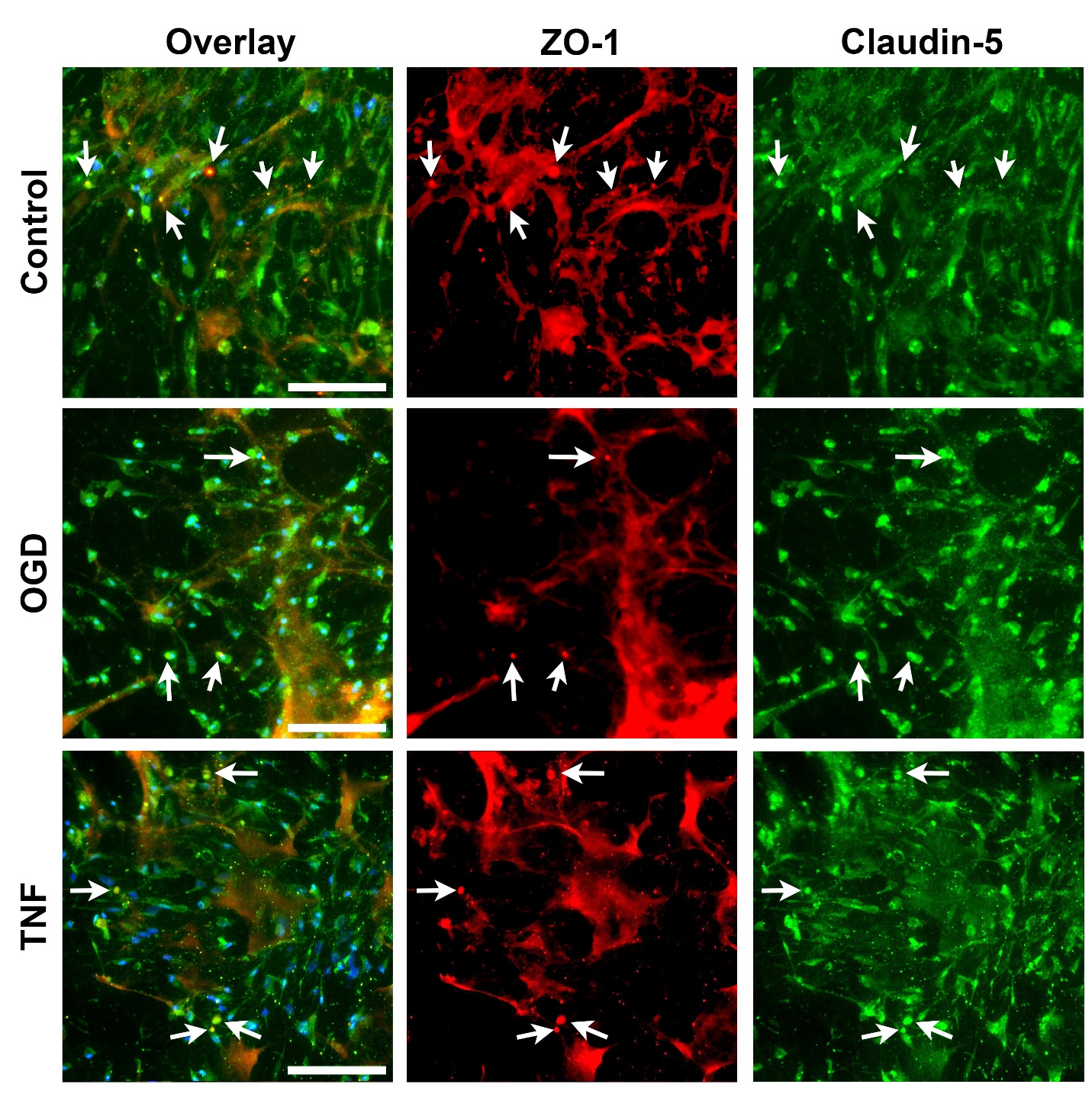


**Supplementary Figure 2**. **Claudin-5 and zo-1 arrangement in contact tricultures under homeostatic, inflammatory (150 ng/ml TNF-α), and metabolic insult conditions.** Contact tricultures were treated with either TNF-α or exposed to an oxygen-glucose deprived environment for 4 hrs before fixation. The TEER of this TNF-α treated triculture was approximately 339.5 Ωcm^2^. TEER of OGD-exposed contact tricultures was approximately 261.3 Ωcm^2^. Arrows represent areas of co-staining between claudin-5 and zo-1. DAPI was used to stain nuclei of all cells in Transwell insert. Scale bar represents 100 µm.


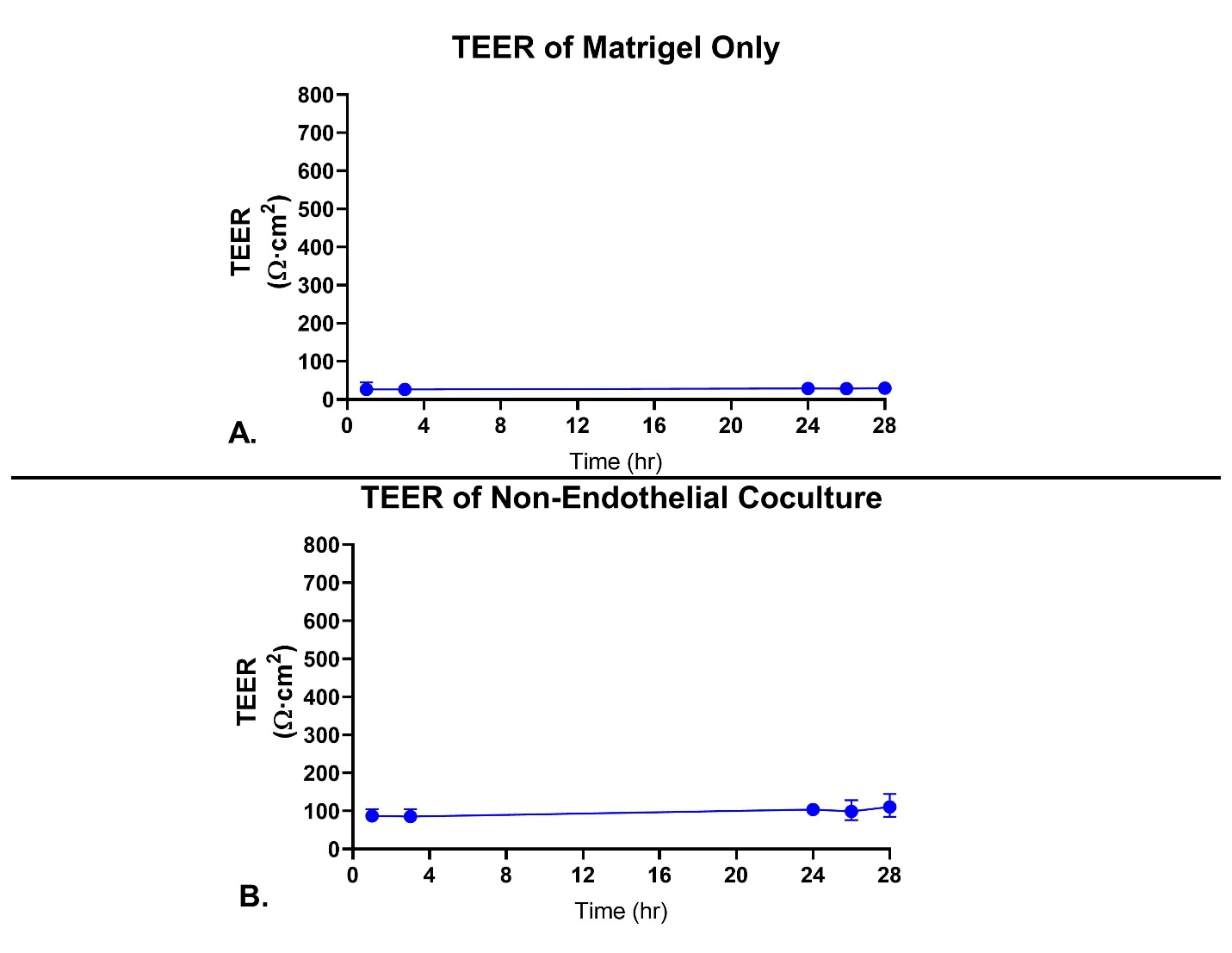


**Supplementary Figure 3. Transendothelial electrical resistance of Matrigel-coated transwells and non-endothelial coculture. (A.)** Matrigel-only coated transwells were a. TEER was measured for a total of 28 hr after addition to the platform. Data is presented as SEM. **(B.)**  Confluent cocultures of primary human astrocytes and BM-MSCs were established on the upper membrane of Transwell inserts. TEER was measured for a total of 28 hr after addition to the platform.


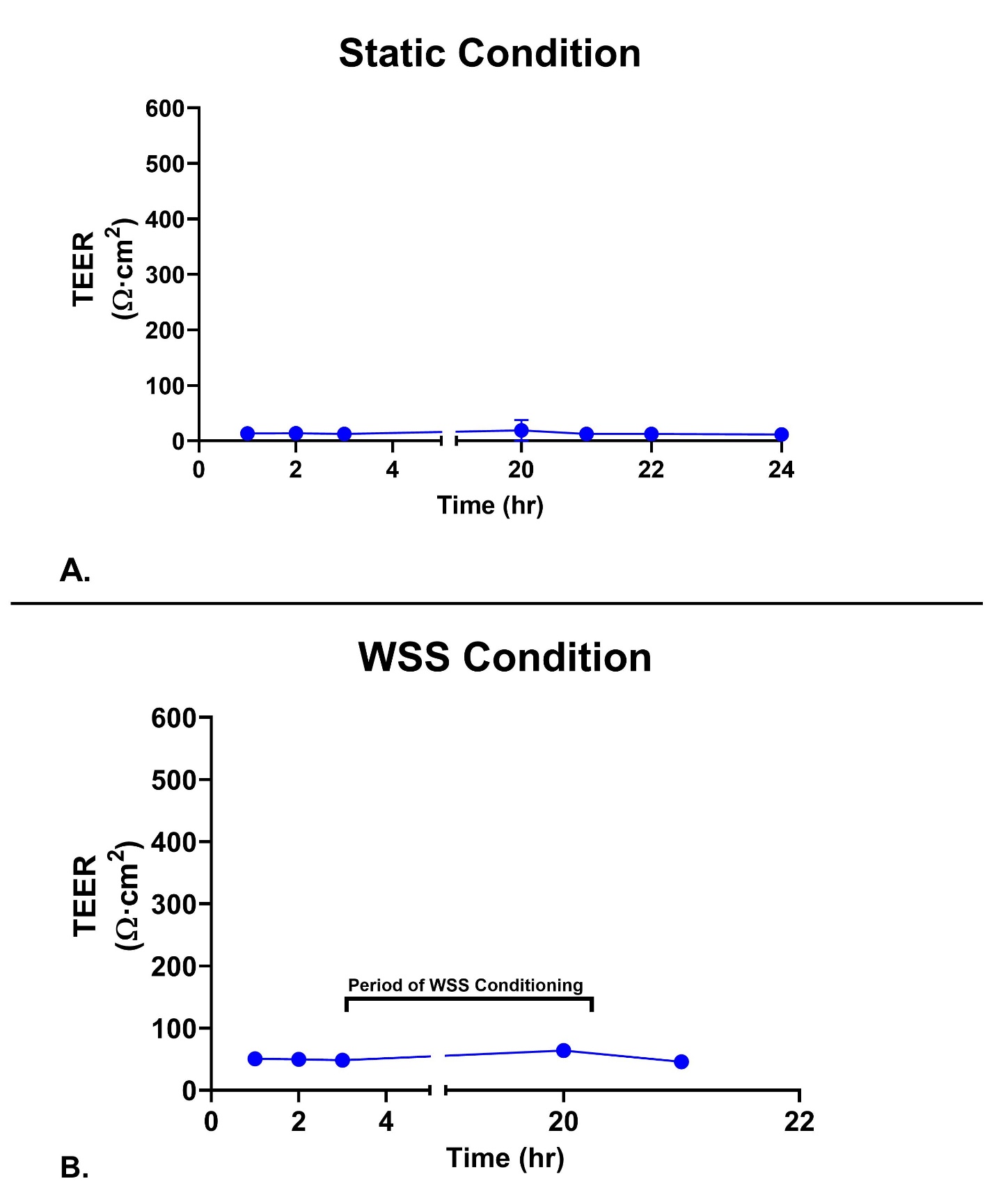


**Supplementary File 4.** **TEER measured following growth under static and dynamic conditions on Matrigel**. **(A.)** Confluent cultures consisting of HUVECs were established on the upper membrane of Transwell inserts. TEER was measured for a total of 24 hr after addition to the platform. Data is presented as SEM. **(B.)** Shear stress was applied for 18 hr after a 3 hr acclimation period to the platform. Confluent cultures consisting of HUVEC (blue circle) were established on the upper membrane of Transwell inserts and allowed to acclimatize to the system for 3hr. We then initiated WSS (4 dyne) for 18hr (t=3hr until t=20hr) followed by additional TEER measurements (t=20-24hr.) Data is presented as SD. **P*<0.05; ***P*<0.01; ****P*<0.001; *****P*<0.0001 (Ordinary One-way Anova). N = 24 (3 repeated experiments of n =8 biological replicates)

**
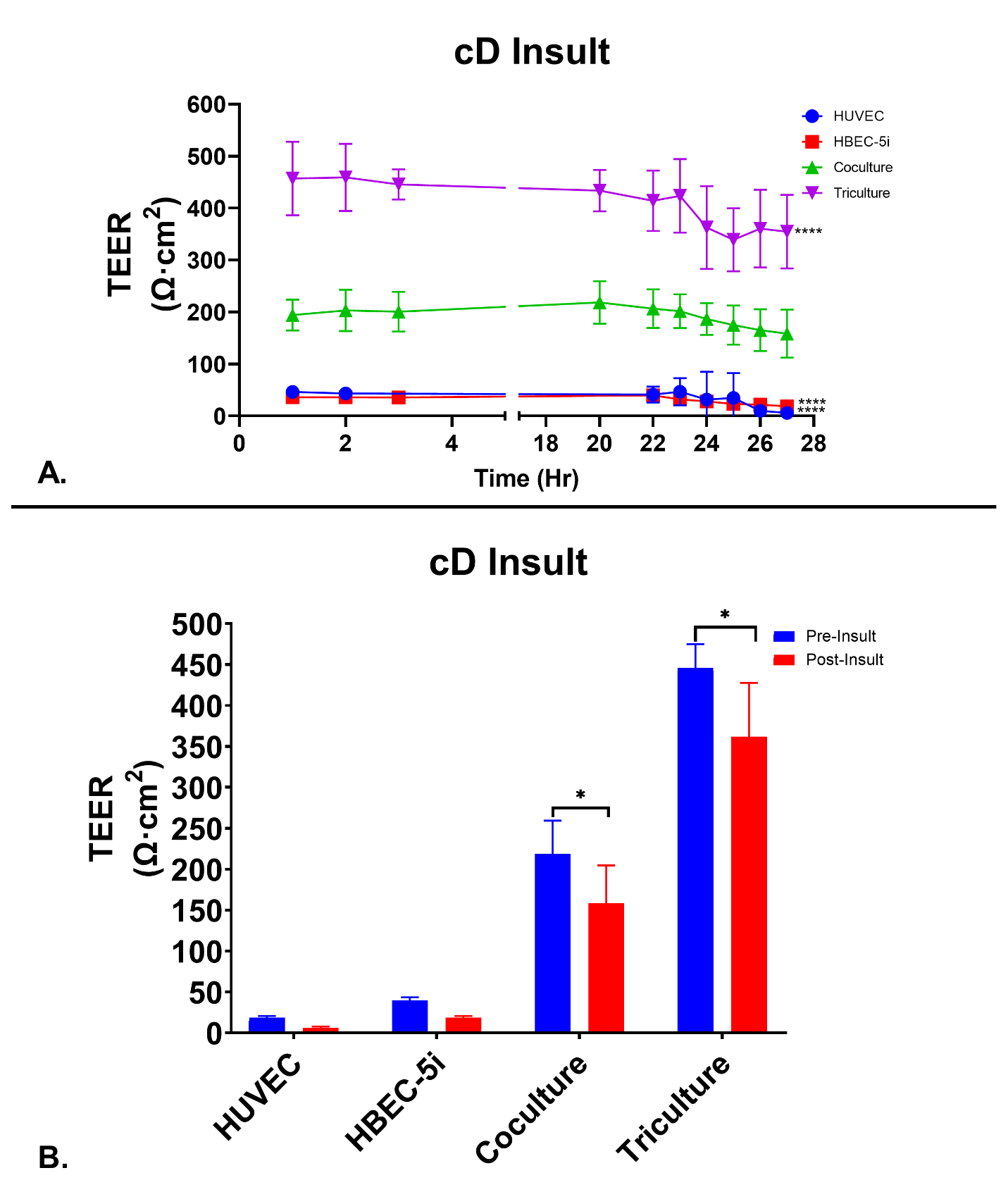
**

**Supplemental Figure 5. cytochalasin-D (2.5 µg/ml) induced changes in barrier properties of endothelial cell constructs.** (**A**.) Confluent cultures consisting of HUVEC (blue circle), HBEC-5i (red square), co-cultured HBEC-5i and primary human astrocytes (green triangle), and tricultured HBEC-5i, primary human astrocytes and BM-MSCs (purple inverted triangle) were grown to confluence on Matrigel coated Transwell filter inserts and added to platform. All endothelial cell monolayers were allowed to acclimate in platform under low flow condition for 20 hr. Following cD treatment at the 22 hr timepoint, TEER measurements were performed every hr for a total of 5 hr. (**B**.) Pre-insult corresponds to hr 20 after cultures were under low flow conditions. Post-insult corresponds to 5 hr after cytochalasin exposure (Hr 27). Data is presented as SD. **P*<0.05; ***P*<0.01; ****P*<0.001; *****P*<0.0001 (Ordinary One-way Anova). N = 24 (3 repeated experiment of n =8 biological replicates)


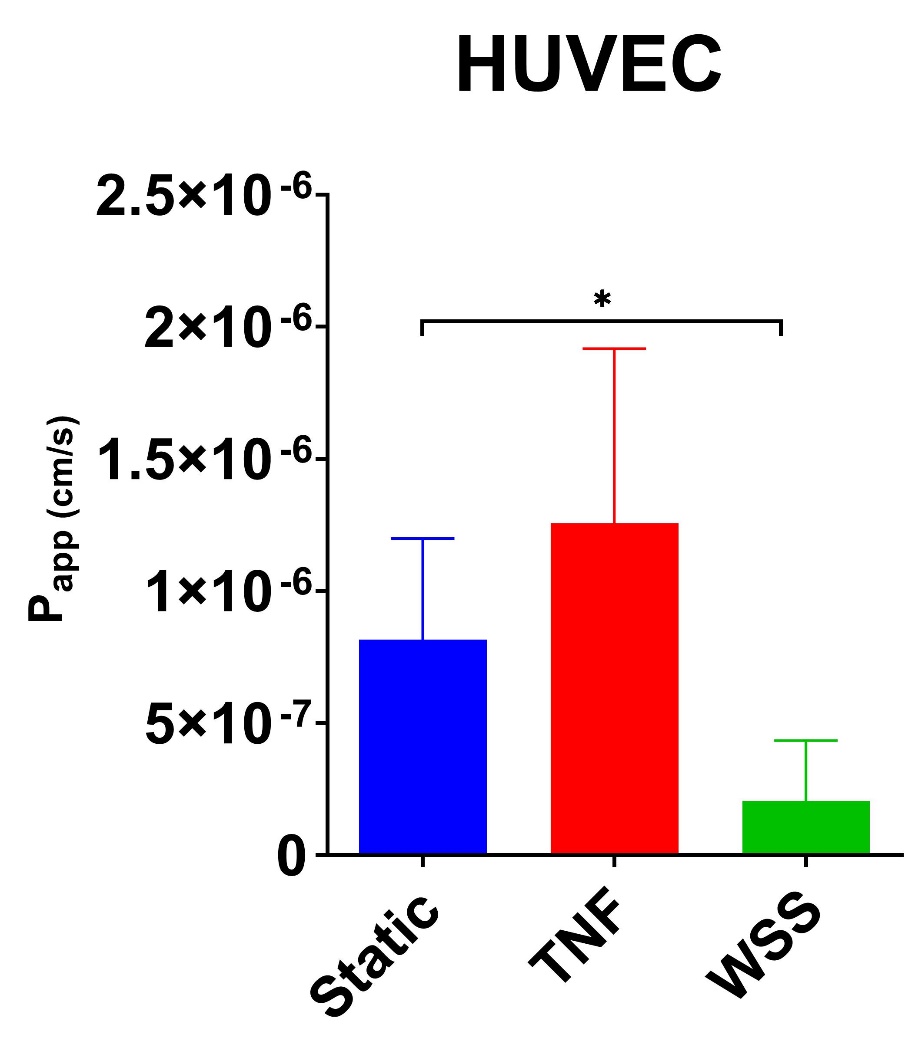


**Supplementary Figure 6. Mean P_app_ values of variable sized tracers in HUVEC monolayers constructs.** The permeability coefficient of 15 kDa FITC-Dextran after static condition, the application of WSS, and treatment with proinflammatory cytokine TNF-α in HUVECs. Data is presented as SD. **P*<0.05; ***P*<0.01; ****P*<0.001; *****P*<0.0001 (Ordinary One-way Anova). N = 24 (3 repeated experiment of n =8 biological replicates).


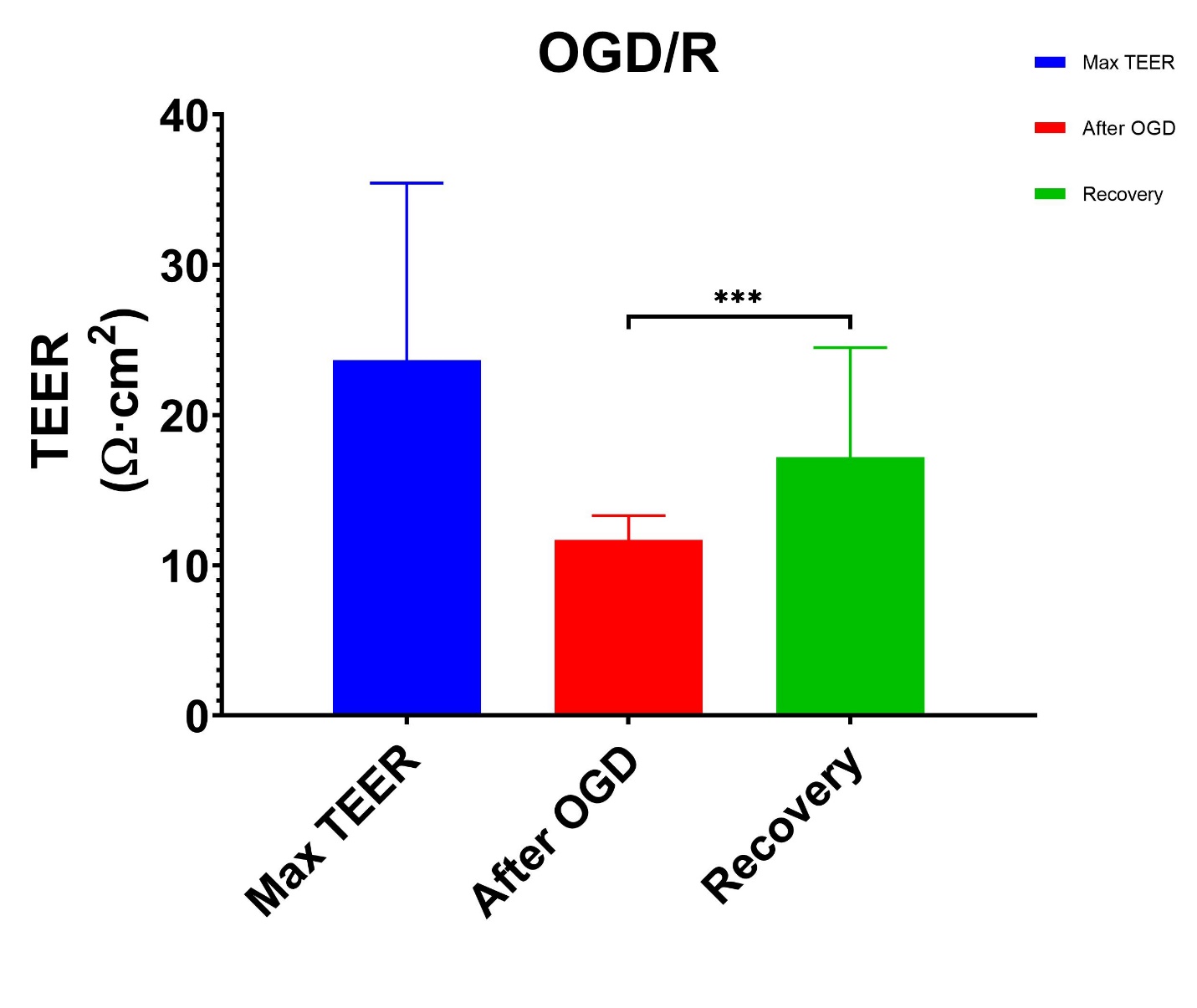


**Supplementary 7. Effects of an oxygen and glucose deprived environment on TEER**. Confluent cultures consisting of HUVECs were established on the upper membrane of Transwell inserts and allowed to acclimatize to the system for 20 hr (Max TEER). After 4 hr of exposure to an OGD environment (After OGD), monolayers had developed a significant decrease in their optimal resistance. Monolayers were reperfused with original growth media and allowed to recover for 24 hrs (recovery). Data is presented as SD. **P*<0.05; ***P*<0.01; ****P*<0.001; *****P*<0.0001 (Two-way Anova). N = 24 (3 repeated experiment of n =8 biological replicates).

| **Expressive Markers** | **Mesenchymal Stem Cells** | **Pericytes** |
| --- | --- | --- |
| **NG2+** | X | X |
| **PDGFRb+** | X | X |
| **CD13+** | X | X |
| **αSMA+** | X | X |
| **CD146+** | X | X |

**Supplementary Table 1. Immunophenotype Similarities between Pericytes and Mesenchymal Stem Cells.**

**Methods**

Reagents

HBEC-5i were initially cultured and maintained in Gibco Dulbecco's Modified Eagle Medium: Nutrient Mixture F-12 (DMEM/F-12) with 10% FBS, 40 µg/mL endothelial growth supplement (ECGS) and 1% penicillin/streptomycin (P/S; ScienCell Research Laboratories, Inc., Carlsbad, CA, USA) in 75 cm^2^ flasks coated with 0.1% gelatin. Primary human astrocytes (PHA) were expanded and maintained in Astrocyte Medium, which contains 500 mL of basal medium, 10 mL of FBS, 5 mL of astrocyte growth supplement (AGS), and 5 ml of P/S in 75 cm^2^ flasks containing a solution of poly-L-Lysine (2 μg/cm^2^). PHA were used between passages 3-5. Bone marrow derived MSCs (BM MSCs) were isolated from commercially available fresh human bone marrow aspirates (AllCells, Emeryville, CA) or obtained from fresh bone marrow through the approved IRB protocol HSC‐MS‐08‐0393 and expanded following established procedures (PMID: 28233425). Donor 5204, used in the current study, was from a 28 years old male. Briefly, BM MSCs were cultured in complete culture medium that consisted of alpha‐minimal essential medium (Life Technologies, Grand Island, NY), 17% fetal bovine serum (FBS; lot‐selected for rapid growth of MSC; Atlanta Biologicals, Norcross, GA), 100 units/ml penicillin (Thermo Fisher Scientific, Waltham, MA), 100 mg/ml streptomycin (Life Technologies), and 2 mM l‐glutamine (Thermo Fisher Scientific, Waltham, MA). BM-MSCs were used between passages 4-5.

*Contact Tri-culture (Chamber slide)*

On day 1, Lab-tek chamber slides coated with attachment factor solution (250 μL) for 30 min.). A 500 μL cell suspension (1.15 x 10^4^) of HBEC-5i in endothelial cell medium (ScienCell) was added to the chamber slide and allowed to grow uninterrupted until day 3. On day 3, media was removed from the chamber slide, and 250 µL of primary human astrocyte cell suspension (2.5 x 10^4^). On day 3, a 100 μL (5.0 x 10^3^) cell suspension of BM-MSCs were added to the chamber in MSC medium (ScienCell).

**Immunostaining - Celltracker staining**

Contact Triculture were established in Lab-tek II chamber slides and fluorescently labeled to demonstrate cellular interactions. CellTracker ™ Red CMPTX was used to label BM-MSCs, CellTracker™ Green CMFDA (ThermoFisher Cat. C2925) was used for primary human astrocytes and HBEC-5is were left unstained. The CellTracker dyes were solubilized in DMSO to a concentration of 10 mM and then further diluted in serum free culture medium (DMEM) to create a working concentration of 2 µM. HBEC-5is were seeded on Lab-tek chamber sides for 48 hrs. before the addition of any other cell types. Primary human astrocytes and BM-MSCs in separate tubes were centrifuged at 1000 x g for 5 minutes to pellet the cells. The resulting pellets were reconstituted in 10 mL of the 2 µM working dye and added to a 6 well plate for incubation. The well plate was then incubated at 37°C in 5% CO_2_ for 30 minutes. After incubation, the dye/cell suspensions was transferred into a tube, centrifuged at 1000 x g for 5 min, and the pellets were washed in 3 mL of DMEM.

**Cytochalasin D Treatment**

Cytochalasin D (cD) from *Zygosporium mansonii* was used to study effects on TEER. Stock solution of cD (5 mg/ml) was made with DMSO and diluted in EGM-Plus before addition to cells. The final concentration of DMSO did not exceed 0.01%. TEER was measured every hour for a total of 5 h after addition of cD (2.5 ug/ml).

**Dextran permeability assay**

Permeability assays were performed using 4 kDa fluorescein-isothiocyanate dextran (ThermoFisher), 10 kDa Alexa Fluor 680, and 40 kDa Tetramethyl dextran (ThermoFisher) in triculture as described previously^35^. All models were assessed in 15 kDa fluorescein-isothiocyanate dextran. After the period of experimental observation concluded, FITC dextran was added to the luminal side to evaluate paracellular transport. The permeability coefficient (P_app_) in the abluminal side was measured by a microplate reader at an excitation of 490 nm and an emission of 520 nm. Calculations for permeability coefficients are as described below:

$$Papp= (\left( Absorbance of dextran)\div period of observation in seconds \right))\div((1\div\left( Transwell surface area\times Dextran concentration \right))$$

The calculation of the permeability coefficient (Pe) was calculated as described below:

$$Pe=(\frac{1}{Permeability}of Cellular layer-\frac{1}{permeability}of transwell)\div Transwell surface area$$

**OGD/R**

After acclimation to the platform, cultures were maintained using EGM-PLUS Medium. EGM-PLUS media was replaced with glucose-free DMEM for OGD conditioning after acclimation in the platform (Max TEER). To remove oxygen from media, media was degassed inside of a tissue culture hood dedicated for platform operation. Glucose-free DMEM was placed in a cuum bell jar and suctioned until no bubbles were visibly present. Media was flushed with anoxic gas mixture for 15 minutes and immediately placed in an anaerobic pouch until use. Cells were allowed to grow in a hypoxic environment (5% CO_2_ and 95% N_2_) for 4 h (After Insult) with all gas exchange intervals capped. OGD conditioning was terminated by removing glucose-free media and supplementing with EGM-PLUS media. Normal culture conditions were then returned, and cells recovered for 24 h (Recovery).

**Western Blot**

Cell-seeded Transwells were removed from the dynamic platform and washed with ice-cold PBS to remove excess cell media. 300 uL RIPA Lysis buffer with 1x Halt Cocktail Protease Inhibitor (ThermoFisher) was added to the Transwell on ice for 30 minutes. Samples were diluted with 4x LDS sample buffer (Invitrogen, Carlsbad, CA), 1x reducing buffer and then boiled at 85 °C for 10 min. Each sample was loaded into the wells of a NuPAGE Novex 4-12% Bis-Tris gel (Invitrogen, Carlsbad, CA) for gel electrophoresis. Proteins were transferred onto nitrocellulose membranes. Membranes were blocked for 1 h at room temperature with either 5% BSA in TBST. Membranes were then incubated with either occludin (1:50,000), claudin-5 (1:5,000), or GAPDH (1:10,000) in their respective blocking buffer for approximately 2 h at 4 °C. After primary antibody incubation, membranes were washed with TBST and incubated with secondary antibody goat anti-rabbit HRP (ThermoFisher) for 1 hr at room temperature. Original blot edges were clipped before use in the transfer process.

The following antibodies were used: anti-claudin-5 (Thermo Fisher, 35-2500), anti-occludin (Thermo Fisher, 33-1500), anti-zo1(Thermo Fisher, 61-7300), goat anti-rabbit AlexaFluor 488 (Thermo Fisher, A11008), and goat anti-rabbit AlexaFluor HRP (A21428, ThermoFisher).

Supplemental Western Blot

Occludin


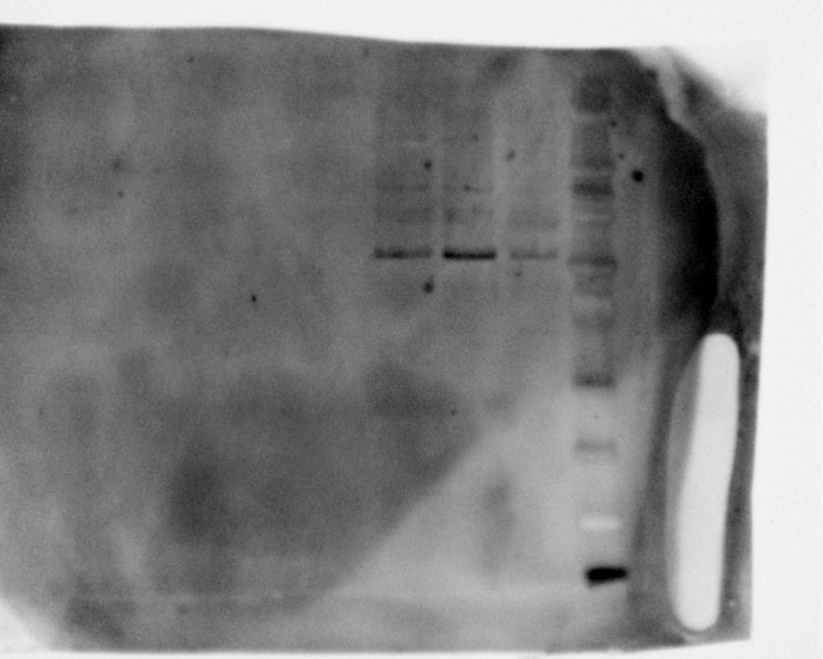


**Supplemental Figure 8. Original Western Blot of occludin**

Claudin 5


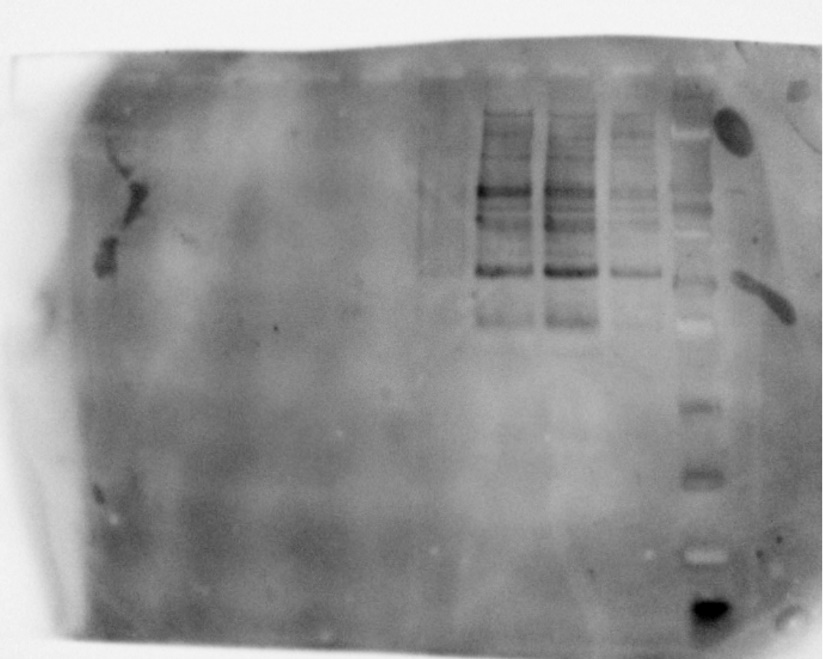


**Supplemental Figure 9. Original Western Blot of Claudin-5**

Zo-1


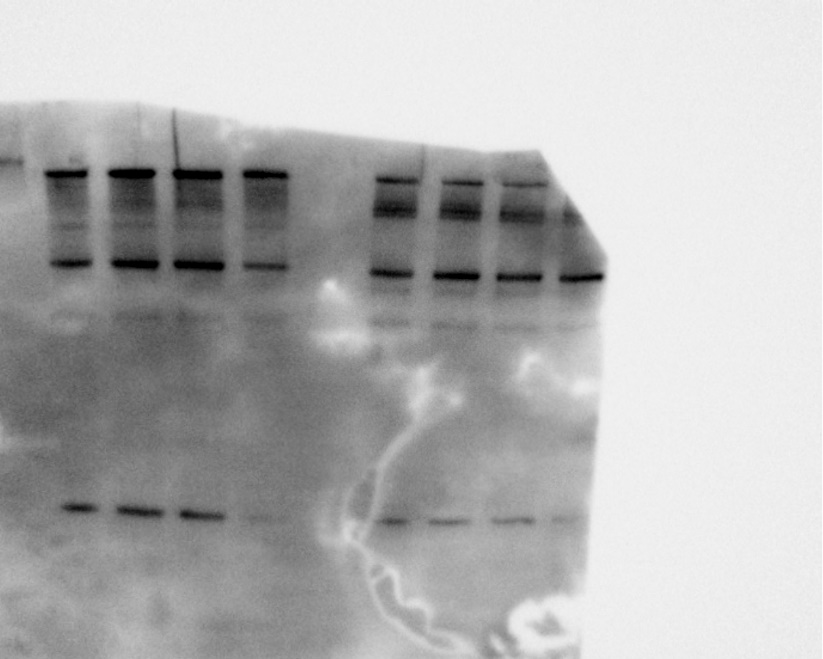


**Supplemental Figure 10. Original Western Blot of Zo-1.**
